# Enhancing computational thinking in early childhood education through ScratchJr integration

**DOI:** 10.1016/j.heliyon.2024.e30482

**Published:** 2024-04-30

**Authors:** Louka Konstantina, Papadakis Stamatios

**Affiliations:** Department of Preschool Education, University of Crete, Rethymnon, 74100, Crete, Greece

**Keywords:** Computational thinking, Coding skills, Early childhood education, ScratchJr

## Abstract

A plethora of programming platforms purports to teach preschool-aged children computational thinking (CT) and coding skills. However, the empirical evidence to support their effectiveness is still in its early stages. A three-week didactic intervention using ScratchJr was conducted to investigate its effectiveness in fostering CT and rudimentary coding skills in a cohort of preschool children (N = 34, aged 4–6 years). While the initial performance mean of the control group (M = 7.07, SD = 2.58) exhibits a statistically significant elevation t (22.64 = 2.23, p = 0.036) vis-à-vis the experimental group (M = 5.35, SD = 1.58), t (22.64) = 2.23, p = 0.0 the terminal performance means of both groups converge closely. However, meticulous data analysis unveils a statistically notable enhancement among preschool participants exposed to the educational intervention involving ScratchJr. Notably, both instructional modalities appear commensurate in nurturing elementary computational tenets, namely modularity and control structures. The experimental cohort outperforms the control group with statistical significance in comprehending potent ideational constructs encompassing representation, algorithms, and hardware/software interplay. Conversely, the control group performs better in grasping the debugging concept than their experimental counterparts. The outcomes lend substantive credence to the efficacy of the distinct programming milieu offered by ScratchJr, underscoring its effectiveness in cultivating CT and coding proficiencies within the preschool demographic.

## Highlights

• ScratchJr boosts CT in preschoolers.
• Empirical evidence supports effectiveness.
• Statistical significance in skill enhancement.

## 1 Introduction

Tomorrow's society and the modern labour market need citizens who are competent problem solvers [1]. In Wing's [2] view, computational thinking (CT) is a problem-solving skill necessary for all citizens. Cultivating CT skills from kindergarten onwards could provide young learners solid groundwork for embracing a systematic problem-solving methodology [3]. Such an approach sets the stage for equipping upcoming generations with the aptitude required to fulfil the requisites of contemporary society. Utilizing programming as an educational tool emerges as an effective strategy for nurturing CT, supported by studies highlighting the feasibility of imparting fundamental programming notions and proficiencies to young children [4,5].

In recent years, apps for smart mobile devices have been developed to introduce preschool children to the basics of programming [6]. Young children begin their first exposure to digital technologies using smart mobile devices in the family environment. By imitating and observing, kids successfully interact with portable digital devices, even if they have not mastered reading and writing [7]. However, choosing the right programming environment is challenging for parents and educators [8] due to the chaotic number of applications that have flooded the unregulated app market [9]. Many apps that claim to be educational have only entertainment content without providing any real value to their users [10]. The present research aims to add to the literature by demonstrating whether the programming environment ScratchJr can cultivate basic programming concepts for preschool children in the context of the Greek kindergarten through a three-week educational intervention.

### 1.1 Computational thinking conceptual approach

The term “computational thinking” is not new. Papert first used it in the 1980s to put children's relationship with electronic computers into a new context [11]. However, the term became fashionable after the publication of the article “Computational thinking” by Jeannette Wing, professor of computer science, in March 2006. Although computational thinking (CT) has been extensively discussed in recent years, there is a lack of consensus on what a definition of computational thinking might include [[12], [13], [14], [15]]. In their research, Román-González et al. [16] have distinguished three categories of definitions of CT. General definitions are the first category. In this category, there is also the definition by Wing [2], who describes CT as a set of mental processes aiming to solve different problems with the parallel participation of the human and mechanical elements. In Wing's view, CT is a global skill everyone uses, not just researchers and scientists. The second category is the operational definition of CT. In 2009, the International Society for Technology in Education (ISTE) and the Association of Computer Science Teachers (CSTA) organized a survey to formulate a working definition of CT. In their operational definition, CT is the extended set of skills designed to solve practical problems and support the behavioral and attitudinal practices associated with computing [15]. The final category is the pedagogical or didactic definition, which includes frameworks for cultivating computational thinking (CT) in classrooms. Brennan and Resnick [17] proposed a three-dimensional framework for computational thinking that includes concepts, practices, and perspectives. The first dimension refers to using concepts in programmers' language, the second dimension refers to the methods and strategies followed during the programming process, and the third relates to students' inner sense of completeness about the technological world.

Denning [18] posits that Computational Thinking (CT) encapsulates a set of abilities that closely align with the skills inherent in programming and problem-solving with computers. In contrast, Bers [19] broadens the conceptualization of Computational Thinking (CT) by delineating it as a process oriented toward expression and creation. This characterization extends beyond the conventional definition of CT as a mere collection of problem-solving actions. Furthermore, she advances a framework that encapsulates seven fundamental facets of CT tailored for preschool children, including algorithms, modularity, representation, control structures, hardware/software interplay, debugging, and the design process. The notion of “powerful ideas,” as initially articulated by Papert [11], is invoked here to allude to competencies within a given domain that confer both personal utility and novel approaches to problem resolution. Within the context of CT, these competencies encompass algorithmic thinking, abstraction, the capacity to deconstruct solution strategies, the identification of patterns and generalization, as well as the cultivation of evaluation and logical reasoning skills, as asserted by Bell et al. [20]. The latter scholars advocate the cultivation of CT within educational contexts that do not necessitate electronic devices.

### 1.2 Computational thinking and programming

**Fig. 1:**
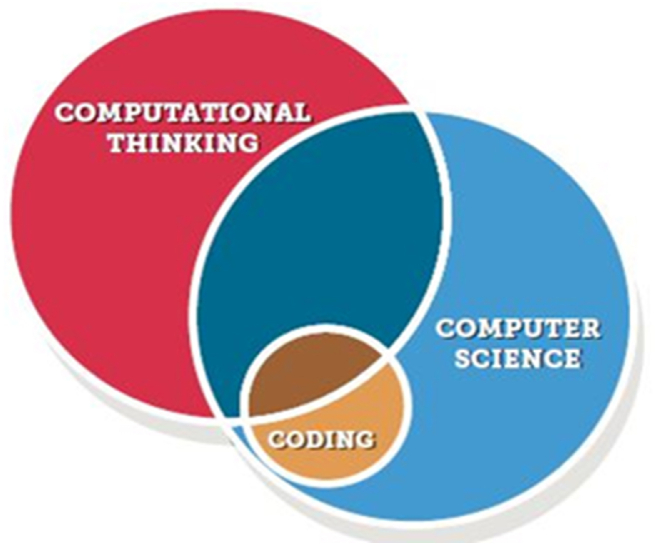
The relationship between coding, computer science and CT [24].

Computational thinking stands as a foundational skill crucial for 21st-century citizens [2]. Bers et al. [21] contend that acquiring programming skills is an effective means of fostering computational thinking. Fig. 1 illustrates the interconnection between computational thinking (CT), computer science, and computer programming. The Association for Computer Machinery (ACM) defines the study of computers and their algorithmic operations as computer science [22]. Notably, computer programming constitutes just one facet of the broader field of computer science [23]. Programming serves as the medium for implementing algorithms on computers, where an algorithm is a meticulous, step-by-step description of a solution to a problem [22]. The act of formulating a set of instructions comprehensible and executable by a computer is referred to as coding [24]. Several fundamental programming concepts have particular relevance to the development of CT in early learning, even though programming is only one aspect of the large field of computer science. Algorithms, or command sequences where the order is important, and control structures, or teaching commands that address how algorithms behave, are arguably the most pertinent examples [25].

Research has demonstrated that the acquisition of CT skills in children may be enhanced by learning to code, possibly due to the fact that coding necessitates logical reasoning which depends on the utilization of sequence and structure [[26], [27], [28]]. Coding and computer programming resources, curricula and activities that foster logical thinking and sequencing are the most widely used and accessible methods for educators to initiate the development of CT in children [23].

Correcting a common misconception that CT is synonymous with coding is essential at this point. While coding is the ideal context for fostering CT, CT is all the mental skills an individual applies to understand and solve a problem predating coding [28]. Voogt et al. [29] come to the same conclusion, arguing that CT goes beyond programming as it is a creative expression that leads to new cognitive pathways. Learning to code is the “new literacy of the 21st century” and the ideal way to teach CT, according to Bers [19].

### 1.3 Programming for the preschool age group

By creating the first children's programming language, Logo, to help young students learn mathematics, Papert [11] pioneered the value of programming in children's education. More recent empirical research has concluded that preschool children can cultivate basic computer science skills through coding using a variety of programming environments [5]. The review of empirical research on early childhood education was based on the classification system of educational programming environments for primary education by Fessakis et al. [30].

The first category, often called “floor turtles”, includes environments based on the Logo programming language. The programmable floor robot Bee-Bot is part of this category. Research using the Bee-Bot floor robot as a tool for developing CT [[31], [32], [33]] shows that children from preschool age, when involved in interventions with this floor robot, master basic programming concepts such as sequencing, detecting and correcting errors, breaking down complex activities into smaller tasks, among others. In addition, mathematical concepts such as the measurement process [31] and spatial relationships and orientation [31,32] have been cultivated.

The second category is educational environments using visual programming interfaces such as ScratchJr. The research conclusions generated by ScratchJr are not limited to the acquisition of CT skills such as sequence comprehension, debugging, symbol interpreting and targeted programming [34]. They also include cultivating creativity in young students [35], making them problem solvers rather than content consumers [36]. In addition, the involvement of children in learning to-code activities activates young students' higher-order cognitive abilities [3].

Programming environments that lack pioneering features and are commercial products fall into the third category. In addition, these environments are a cause for concern as they do not support socially equitable access to knowledge [30]. Results from studies using commercial programming learning environments like Kodable show that preschoolers can successfully learn basic programming skills, including sequencing and debugging [37].

Bers et al. [21] research has shown that integrating learning-to-code activities through robotics kits is possible in preschool curricula—physical computing environments [30]. The KIBO educational robotics kit belongs to the fourth category of the classification system. Studies on using KIBO with teaching activities aimed at children's understanding of basic CT concepts have had positive results [21,[38], [39], [40]]. Research has found that the KIBO robot and some aspects are suitable for 3-year-olds despite being designed for 4-year-olds and older [21,38,39]. There is also emerging evidence that robotics/programming supports music and dance learning [21,40].

Limited attention has been directed towards the integration of Computational Thinking (CT) into formal education, particularly within the context of early childhood education [5]. Nonetheless, the cultivation of CT has garnered substantial interest as a skill deemed valuable for the entire student demographic [2]. In a comprehensive survey of relevant scholarly works, Lye and Koh [27] observed a paucity of empirical investigations, with only two studies identified that specifically address the instillation of CT in preschool-aged children through coding instruction. Furthermore, Macrides et al. [5] concur that the incorporation of coding at the preschool level remains at an incipient stage, and a dearth of empirical research concerning the inclusion of coding in the compulsory kindergarten curriculum continues to persist, necessitating further scholarly inquiry.

### 1.4 ScratchJr for the development of computational thinking in preschool education

**Fig. 2:**
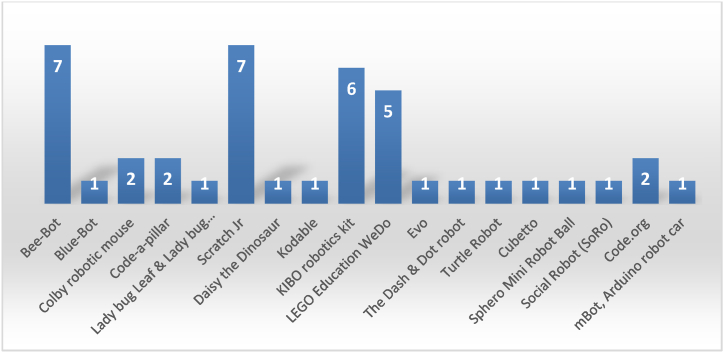
Popular environments for programming [41].

Developmentally appropriate programming environments are the cornerstone of successful CT and coding integration [5]. Louka's [41] systematic literature review highlighted developmentally appropriate programming environments for early childhood education. As shown in Fig. 2, ScratchJr is a well-known programming environment for developing essential CT and coding concepts and was used in seven reviewed studies with positive results. The Bee-Bot floor robot proved to be a prevalent programming environment after it was used in seven studies to cultivate computational thinking in kindergarten. The KIBO robotic kit was selected in six research papers to evaluate its effectiveness in developing introductory programming concepts. At the same time, five studies assessed the LEGO WeDo robotic kit for its efficacy in promoting CT skills (see Fig. 2).

ScratchJr is a visual programming environment which allows a young age user to create programs by connecting appropriate blocks of code [42]. ScratchJr is an age-friendly and easily manageable programming language that empowers experimentation, leads to the construction of new knowledge, and allows the cultivation of complex concepts. It allows creation and exploration and has a user-friendly interface that positively enforces learning [43]. It is designed as a “coding playground” [21]. The confluence of these attributes renders ScratchJr a programming environment that aligns with developmental appropriateness. It is on the basis of these articulated rationales that ScratchJr has been selected as the platform for the execution of the present research.

Recent investigations have established that preschool-aged children can cultivate Computational Thinking (CT) skills through engagement with the ScratchJr programming environment. A comprehensive systematic review of the literature, as conducted by Louka et al. [44], identified a corpus of seven empirical inquiries, each of which arrived at the consensus that the ScratchJr application is efficacious in enabling preschool learners to grasp fundamental CT concepts and acquire proficiency in coding within the kindergarten classroom setting. These studies, authored by Strawhacker et al. [34,35], Falloon [3], Papadakis et al. [36], Portelance et al. [45], and Flannery et al. [43], encompassed four surveys carried out in the United States, one in New Zealand, and one in Greece.

The synthesis of prior research underscores the ability of children aged 4 to 7 to proficiently grasp fundamental programming concepts through appropriately tailored instructional approaches. For instance, the investigation by Flannery et al. [43] has demonstrated that introducing young children to programming environments attuned to their developmental stage yields substantive improvements in programming proficiency. In a similar vein, Papadakis and his research associates examined the impact of employing ScratchJr as an instructional tool to impart basic programming concepts and foster Computational Thinking (CT) within the preschool educational context [36]. This study, in particular, revealed that preschool-aged children could adeptly employ ScratchJr command blocks and leverage them to construct intricate functional projects.

Furthermore, the study conducted by Strawhackers et al. [34] delved into the interplay between teaching methodologies employed by educators and the resultant learning outcomes from interventions aimed at cultivating elementary programming concepts. Portelance et al. [45] explored the command block choices made by young children while crafting their projects using ScratchJr. In a complementary vein, the investigation by Strawhacker et al. [35] inquired into the specific cognitive domains that children engaged with during their programming activities with ScratchJr, as well as the types of errors they encountered when their coding proficiency was assessed. Lastly, Falloon [3] delved into the examination of the broader cognitive reasoning abilities exhibited by children as they embarked on their journey to acquire programming skills.

In most of the studies reviewed, the age range of participants varied between 5 and 7 years, including children in preschool and the first two years of primary school. In all other studies, the intervention was delivered in the context of a physical classroom, except for the study by Flannery et al. [43], where part of the first phase was delivered as a summer programme. In the studies reviewed, the educational intervention was long-term. Regarding research design, four studies combined qualitative and quantitative approaches to analyzing data [3,[34], [35], [36]]. Furthermore, during the design of the digital application ScratchJr, officially released for the Apple iPad in 2014, an observational pilot study was conducted by Flannery's research et al. [43], while a quasi-experimental design was conducted by Portelance et al. [45]. Falloon's research [3] explored using ScratchJr to teach basic shapes. The DevTech research team designed Animated Curriculum Genres used in most studies to engage participants in activities [45].

Regarding outcomes, children learned ScratchJr app features and commands across interventions and understood the concept of sequencing. The sequence is an essential preschool skill and the foundation of the design process, involving placing objects or actions to achieve a goal [19]. Preschoolers also developed skills in identifying and correcting code errors, interpreting symbols and goal-directed programming in the Strawhacker et al. [34] study. Researchers also observed statistically significant differences between children across classes: the older the participants, the more significant the performance improvement. Similarly, Strawhacker et al. [35] found that preschoolers had difficulty understanding digital flow control blocks and managing more than one character [36]. For older children, these concepts were more accessible, and the focus was on engagement with more complex processes. Only some children needed help understanding the most fundamental programming concepts after the training. These findings echo those of Portelance et al. [45], which found differences in how students performed after analyzing the data. Grade 2 children used significantly fewer end and start blocks than kindergarten children. Older children also used more flow control blocks than their younger counterparts. The researchers identified specific blocks that were challenging to comprehend.

Despite this, the research did show that all of the children showed a particular preference for the use of the movement blocks. The children showed particular interest and enjoyment [36,43] during their programming activities, being active when engaging with these new learning experiences. Instead, participants were actively solving problems [36,43]. This finding is consistent with Falloon's [3] study, which found that higher forms of thinking are practiced in preschool education while engaging in code learning activities.

In summary, the rationale underlying the necessity for the proposed research can be encapsulated through the identification of the following key deficiencies and imperatives.

• The age range of the sample that participated in the reviewed surveys. The age range of participants varied between 5 and 7 years [[34], [35], [36],^43^,^45^]. Τhe present research involved children aged 4 to 6.
• The incomplete design of the ScratchJr application [43,45]. After a decade since its initial release, the application remains in its beta version.
• The time intervals that elapsed until the completion of the intervention. There was no continuous flow in the intervention sessions as the frequency of their implementation could vary from twice a week [35,36] or once every three to four days [36].
• The different teaching styles of the teaching staff. The approach of the teaching staff in promoting social/behavioral skills differed [36] and there were variations in teaching practice as teachers were given freedom to make changes to the curriculum [45].
• The lack of an empirical study with an educational intervention comparing the effectiveness of ScratchJr and unplugged activities in fostering computational thinking (CT).
• The lack of a short-term educational intervention using ScratchJr to foster computational thinking (CT) in preschool education.

There exists a demand for comprehensive research on this matter, as underscored by the findings of the literature review, and this necessity is compounded by the constraints inherent to the methodological approach within the reviewed research. The primary objective of this particular study is to explore the potential of the ScratchJr programming environment in facilitating the acquisition of basic programming concepts in preschool children, specifically those aged 4–6 years, within the framework of Greek kindergarten settings. This inquiry will be carried out through a three-week educational intervention, which will be delivered directly by the researcher, who also serves as the educator, within the formal classroom environment. The proposed research will address the following research questions.

• Does a short-term educational intervention using the app ScratchJr promote CT in preschool children?
• Through a short-term educational intervention using the ScratchJr app, what basic CT concepts will preschoolers develop?

## 2 Materials and method

### 2.1 Sample

The sampled population consisted of preschool children aged 4–6 attending a public kindergarten in a semi-urban area of the Prefecture of Heraklion, Crete, Greece. The sample was selected using non-probability (convenience) sampling. The researcher's connections with the kindergarten staff and parents helped build trust and rapport, essential for the study's success. The initial sample included 36 children, 18 boys and 18 girls. The head teacher completes allocating children to classes at the beginning of the school year. The intention is to have the same number of children in the classes, taking into account gender and age group. The sample has two kindergarten classes with the same number of preschool children. The final sample included 17 students in the experimental group and 15 students in the control group. Of the 32 students, 17 were girls and 15 were boys. One toddler refused to participate in the research, and three were absent for health reasons during the educational intervention. The intervention took place in the same school for both groups. The kindergarten had three classes. The class of the teacher/researcher was the experimental group in which ScratchJr activities were used. The second class was the control group, in which an identical intervention was carried out but with “unplugged” activities, i.e. “disconnected” from the computer. The control class had another regular teacher. The control group children were moved to the researcher's classroom so that the researcher herself could implement the intervention.

### 2.2 Intervention procedure

A quasi-experimental design with an experimental group and a control group, each of which received a pretest and a posttest, was used to conduct this research over three weeks. The intervention occurred during the day in a typical class in kindergarten. Every day, a “powerful idea” was taught within the kindergarten curriculum. The term ‘unplugged’ is used to describe activities that do not require the use of electronic technology [46]. As the proposed research involved data collection from children and was approved by the Research Ethics Committee of the University of Crete, the Code of Ethics for Educational Research with Children was applied throughout the study. Written consent was requested from the parents and guardians of the children who participated in the research after being given a detailed description of the purpose and the procedure. The researcher ensured the anonymity of the participants and ensured the protection of their data. In addition, the participants were informed about all the features of the research and about their voluntary participation in terms that the child could understand [47].

Two assessment tools were used. The TechCheck assessment tool [48] was given to the control group. TechCheck is an “unplugged” assessment tool for CT designed for children between 5 and 9. This tool has three different versions, depending on the age group to which it will be applied. TechCheck-K, suitable for preschool children [49,50], was used in the present study (see Appendix 1 for a sample page). Bers [19] describes it as an ‘unplugged’ puzzle-like coding test consisting of 15 multiple-choice tests exploring six CT domains. Five questions test understanding of algorithms, and two test modularity, control structures, representation, hardware/software, and debugging, respectively, are developmentally appropriate for young children. The design process is also included as a central concept of CT at this point, according to Bers [19]. However, as this is a continuous and open process, a questionnaire with multiple-choice questions cannot assess this, and this research is excluded [48]. The execution of both the pre-test and post-test adhered to the guidelines provided by the questionnaire's creator.

The researcher created an identical questionnaire for the experimental class to assess ScratchJr activities, as TechCheck-K assesses unplugged activities (see Appendix 2 for a sample page). The newly introduced questionnaire was employed for the pre-test. Its utilization had no discernible impact on the subsequent post-test performance of the children. Instead, it induced a heightened level of confusion, attributable to the unfamiliarity of its content. A translation into Greek was carried out due to the foreign language nature of the assessment tool questions. To establish the functionality of the two questionnaires, a convenience sample of four kindergarten children of different ages from the third class of the kindergarten was used to pilot them. Both assessment tools are available as supplementary files. The questionnaire employed in the experimental group can be made available upon request.

**Fig. 3:**
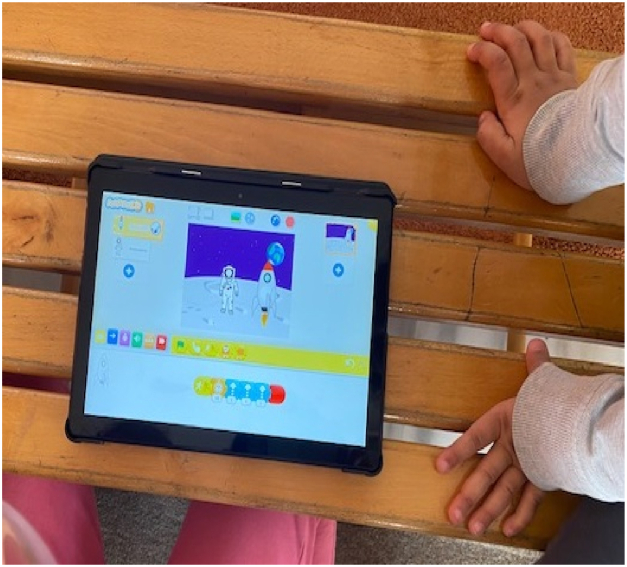
Working with ScratchJr.

**Fig. 4:**
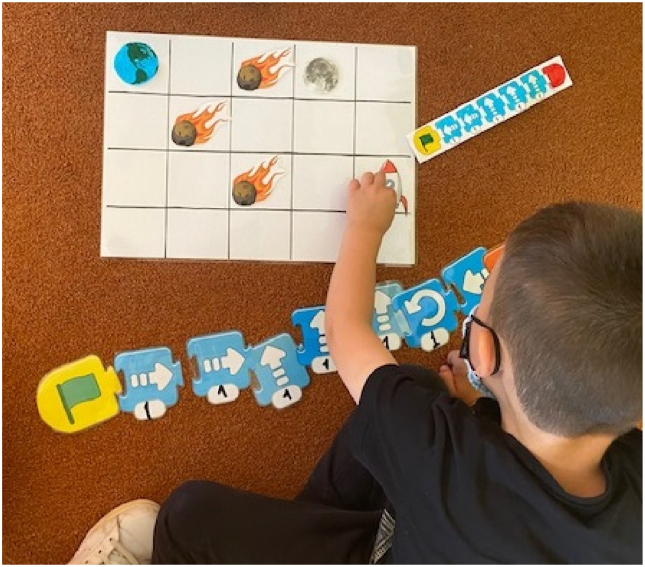
Unplugged activity.

In the initial phase of the study, a pretest was administered within the researcher's classroom. To facilitate this, the preschool teacher, who also served as the researcher, arranged the classroom by placing six small tables within the teaching area. Each table accommodated one child, who was tasked with individually completing a questionnaire. This approach ensured that each child independently responded to the questionnaire, without being influenced by their peers, thereby preserving the integrity of the data. The questionnaire was displayed on the classroom's whiteboard through the use of a video projector. To maintain a seamless projection, the researcher, who was also the kindergarten teacher, configured the classroom appropriately for video projection. The instructions for each question were read aloud to the children twice, and they were allotted 1 min to provide their responses, with an encouragement to make educated guesses if they were uncertain. Once the first group of six children had completed the process, the subsequent groups followed suit until all students had completed the questionnaire. This procedure was consistent for both the experimental and control groups. The subsequent phase of the study, lasting three weeks, involved a teaching intervention conducted in both the experimental and control groups. The intervention protocol was identical for both groups, with children working in pairs. These pairs remained constant throughout the intervention. In the experimental group, each pair of students was equipped with a tablet (see Fig. 3, Fig. 4).

During the third and concluding phase of the study, the researcher, who also served as the educator, instructed both groups of children to once more complete the post-test questionnaire subsequent to the intervention.

### 2.3 Intervention theoretical background

As Bers [19] suggested, the intervention design was based on the seven ‘powerful ideas' of CT for preschool children. The intervention lasted three weeks for both groups. It took place from April 2022 to May 2022. A “powerful idea” was taught daily in the kindergarten curriculum. However, as mentioned above, because the design process is an ongoing, open-ended process that multiple-choice questionnaires cannot measure [48], the “powerful idea” of the design process was taught but not assessed. There were four activities on each day of the intervention.

a) Psychological and cognitive preparation activity for forming the appropriate lesson outline
b) Teaching a ‘powerful idea”.
c) Consolidating the “powerful idea” through problematic situations, and
d) The evaluation and meta-awareness activity, in which the children come together in the classroom, and each group present their work, describing the steps taken in arriving at the final product, pointing out any difficulties encountered, answering questions from the rest of the class and reflecting on their feelings throughout the process. Table 1 presents the activities designed to teach the “powerful idea” of representation. Each activity was 45 min, except for the psychological preparation activity, which lasted 15 min. An introductory activity was designed for the smooth introduction of children to the development of the basic concepts of computational thinking. In the introductory activity, the programmer sends a multimedia file to the children and asks for their help. His friend, the astronaut, needs help. The spaceship's computer malfunctions. Instead of launching and heading to the moon, landed on a beach. Asks children to correctly program the spaceship's computer to go to the moon. Each day, the programmer sent a multimedia file to the children that constituted the psychological preparation activity and introduced them to the teaching of a powerful idea. The activity of this category is related to the formation of an appropriate motivation for the lesson (psychological preparation). The educational intervention's activities can be made available upon request.

**Table 1:** Educational intervention's activities for teaching the “powerful idea” of Representation.

Psychological and cognitive preparation activity	ScratchJr activities	Unplugged activities
	Presentation of multimedia file.	Presentation of multimedia file.
The activity of teaching a ‘powerful idea”:	The researcher presents to the children projects she has created in ScratchJr and encourages the children to observe the code in the programming area and make assumptions about what will happen to the “character”. Next, she changes the order of the blocks and again asks the children to make assumptions about what will happen.	Cards depicting different algorithms. Each team chooses one card. The teams on a squared A4 paper are asked to execute the code shown on the card using a conventional figure of a spaceship.
Consolidation activity	Children, in groups, take their tablets and are encouraged to use the new motion blocks they have learned to create their projects by making the spaceship.	Game in groups of 2 using a squared carpet and cards illustrate the blocks of ScratchJr. Child A will be the programmer, and Child B will be the spaceship. The programmer is the one who creates the code that tells the spacecraft's computer what to do. The code is the instructions. The programmer will have to give the proper instructions to the spacecraft computer to get it to its final destination, the moon. The programmer's instructions are the cards that illustrate the blocks of ScratchJr.
Evaluation and meta-awareness activity	In plenary, groups share the projects they created, are encouraged to show the code they have created, answer questions from their classmates about their work, and are rewarded by everyone for their efforts.	In plenary, each group presents the code and its execution, receiving feedback from the other groups and expressing positive and negative judgments regarding the activities that preceded it.

The design of the intervention incorporated behavioral and socio-cultural theories. Specifically, behavioral teaching strategies, such as information provision and problem-solving, were employed when introducing the ScratchJr programming environment in various activities. To facilitate the acquisition of knowledge about the digital application ScratchJr, probing questions were utilized by the teacher to guide the children. Simultaneously, the teacher aimed to foster active engagement and interest among the students in her instructional approach.

The problem-solving demonstration teaching strategy formed the foundation for addressing more intricate problems that the young students would tackle in pairs during subsequent activities. The social constructivist teaching strategy in the group work activity drew upon socio-cultural theory, emphasizing that children learn through collaborative efforts within small groups. In accordance with social construction theory, the process of solving open-ended problems [51] contributes to the construction of individual knowledge through social interactions. Throughout the activities, the kindergarten teacher assumed the role of a ‘facilitator’ of learning. However, when deemed necessary for the learning process, the teacher also acted as a guide, offering appropriate support where required.

### 2.4 Measurements

The variables that will be the subject of measurement in this research will be.

• Independent variables: The ScratchJr programming environment.
• Dependent variables: as presented in Table 2

**Table 2:** Developmentally appropriate powerful CT ideas for the preschool age group [19].

Powerful idea	Definition
**Algorithms**	The sequence of instructions arranged in a way that leads to solving a problem or achieving a goal, logical organization
**Modularity**	Decomposing a complex task into smaller ones
**Control structures**	Pattern and repetition recognition
**Representation**	Using symbols to represent actions
**Hardware/Software**	Recognizing that technologies are artificial.
**Debugging**	Identifying errors. Developing strategies to resolve them.

## 3 Results and discussion

### 3.1 Results

**Fig. 5:**
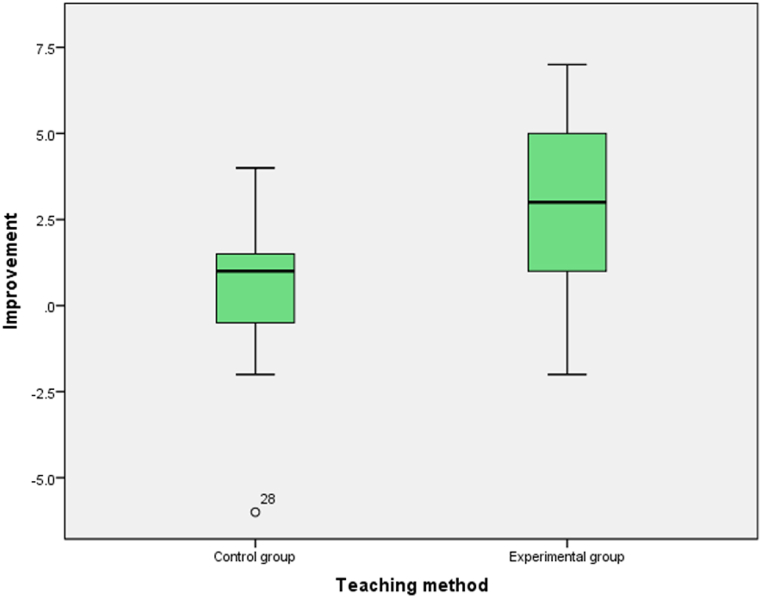
Mean improvement by teaching method after intervention.

We adapted the level of statistical significance in accordance with the sample size, deeming a test result with p < 0.1 to be indicative of statistical significance. This decision was motivated by the relatively modest size of our sample, as advocated by Kim and Choi [52]. It should be mentioned that where the two groups had approximately equal average performance it was deemed necessary to check the average improvement of the two groups after the intervention. When investigating the first research question, the mean performance of the control group (M = 7.07, SD = 2.58) in the pretest was significantly higher than that of the experimental group (M = 5.35, SD = 1.58), t (22.64) = 2.23, p = 00.036. In the posttest, the independent samples *t*-test showed no significant effect of the teaching method, t (30) = −0.83, p = 00.41, in developing CT. However, after the intervention, the experimental group's mean improvement (M = 2.76, SD = 2.44) was found to be statistically more significant, t (30) = −2.8, p = 00.009, than the control group's mean improvement (M = 0.33, SD = 2.47), as shown in Table 3. These results suggest that both teaching methods proved equally effective, with the same average performance. However, data analysis revealed a statistically significant improvement in preschool children whose educational intervention included ScratchJr as the mean improvement was strongly statistically significant with the experimental group being superior by 2.43 points (Fig. 5).

**Table 3:** Summary data analysis of the first research question.

First research question	Control group		Experimental group		p
	Mean	SD	Mean	SD	
pretest	7.07	2.58	5.35	1.58	P < 0.05
posttest	7.40	2.82	8.18	2.46	p > 0.1
Posttest-pretest	0.33	2.47	2.76	2.44	p < 0.01

Concerning gender, an independent samples *t*-test showed that the mean performance of the boys (M = 8.73, SD = 2.46) was weakly statistically higher, t (30) = 1.95, p = 00.06 than the mean performance of the girls (M = 7, SD = 2.55) in the posttest (see Table 4).

**Table 4:** The average performance by gender.

Average performance	Gender	Mean	SD	p
	Boy	8.73	2.46	0.5 < p < 0.1
	Girl	7.00	2.55	

Furthermore, boys (M = 3, SD = 3.02), t (13) = −1.83, p = 00.09, and girls (M = 2.56, SD = 1.94), t (15) = −2.11, p = 00.052, in the experimental group improved, statistically, slightly more than boys (M = 0.14, SD = 3.02) and girls (M = 0.50, SD = 2.07) in the control group (see Table 5).

**Table 5:** The average improvement of boys and girls by teaching method.

Average improvement	Control group		Experimental group		p
	Mean	SD	Mean	SD	
Boy	0.14	3.02	3.00	3.02	0.05 < p < 0.1
Girl	0.50	2.07	2.56	1.94	0.05 < p < 0.1

In terms of age group (see Table 6), the mean improvement of the 5-year-olds (M = 2.28, SD = 3.25) was marginally more statistically significant, t (25.36) = −1.72, p = 00.098, than the mean improvement of the 4-year-olds (M = 0.79, SD = 1.53).

**Table 6:** Average improvement by age group.

Average improvement	Age group	Mean	SD	p

	4 years old	0.79	1.53	0.05< p < 0.1
5 years old	2.28	3.25		

In addition, the mean improvement for the five-year-old preschool children in the experimental group (M = 3.58, SD = 2.11) was statistically more significant, t (16) = −2.88, p = 0.01, than the mean improvement for the control group children (M = −0.33, SD = 3.72) (see Table 7).

**Table 7:** Average improvement of five-year-olds by teaching method.

Average improvement	Teaching method	Mean	SD	p
	Control group	−0.33	3.724	P = 0.01
	Experimental group	3.58	2.109	

Regarding the age group, these findings demonstrate that both teaching methods led to the same mean performance in the posttest. However, the mean improvement was slightly higher for the five-year-old preschoolers than the four-year-old preschoolers. As for gender, boys and girls did not differ in their performance in understanding basic programming concepts, although boys slightly outperformed in this study.

The second research question examined whether a short-term educational intervention using the ScratchJr app would develop the basic concepts of CT in preschool children. Concerning the basic concept of algorithms, the independent samples *t*-test showed that the mean improvement of the experimental group (M = 0.94, SD = 1.14) was statistically more significant, t (30) = 2.73, p = 00.011, than the mean improvement of the control group (M = −0.27, SD = 1.98). Regarding the powerful idea of representation, the mean scores of the experimental group (M = 1,35, SD = 0,49) were statistically more significant, t (30) = -4,59, p = 0,00, than those of the control group (M = 0,53, SD = 0,52). Similarly, the average performance of the experimental group (M = 1.82, SD = 0.39) in teaching the computing concept of hardware and software was significantly higher, t (21.89) = −3.13, p = 00.005, than that of the control group (M = 1.20, SD = 0.68). In terms of the basic concept of modularity, the mean improvement of the experimental group (M = 0.59, SD = 0.80) was approximately equal to the mean improvement of the control group (M = 0.13, SD = 0.74), t (30) = −1.67, p = 00.11. It should also be noted that the mean improvement of the experimental group (M = 1.06, SD = 0.66) in terms of the teaching of control structures was approximately equal, t (21.9) = −0.58, p = 00.57, to the mean improvement of the control group (M = 0.20, SD = 0.41). Of particular interest was the teaching of the computational concept of debugging. The results of the independent samples *t*-test showed that the mean scores of the control group (M = 1,60, SD = 0,74) were significantly better than those of the experimental group (M = 0,76, SD = 0,66) at the post-test, t (21,89) −3,13, p = 0,005. These results indicate that both teaching methods can be considered equal in developing the basic computational concepts of modularity and control structures. The experimental class performed significantly better than the control group in understanding representation, algorithms and hardware/software programming concepts. The control group demonstrated higher performance in understanding the concept of debugging than the experimental group (see Table 8).

**Table 8:** Summary data analysis of the second research question.

	Control group		Experimental group		p
	Mean	SD	Mean	SD	
Average improvement					
Algorithms	−0.27	1.98	0.94	1.14	p < 0.05
Modularity	0.13	1.06	0.59	0.80	p = 0.11
Control Structures	0.20	0.41	1.06	0.66	p = 0.57
Average performance					
Algorithms					
pretest	2.27	1.03	1.35	0.86	p < 0.05
posttest	2.00	1.41	2.29	1.05	p > 0.1
Modularity					
pretest	0.80	0.76	0.29	0.47	p < 0.05
posttest	0.93	0.70	0.88	0.70	p > 0.1
Control Structures					
pretest	1.00	0.88	0.71	0.77	p > 0.1
posttest	1.20	0.78	1.06	0.66	p > 0.1
Representation					
pretest	0.47	0.64	1.00	0.61	p < 0.05
posttest	0.53	0.52	1.35	0.49	p < 0.01
Hardware/Software					
pretest	1.13	0.74	1.35	0.70	p > 0.1
posttest	1.20	0.68	1.82	0.39	p < 0.01
Debugging					
pretest	1.40	0.83	0.65	0.79	p < 0.05
posttest	1.60	0.74	0.76	0.66	p < 0.01

### 3.2 Discussion

Before referring to the results of the data analysis of the first research question, which investigated whether a short-term educational intervention using the digital application ScratchJr could cultivate CT in preschool children (4–6 years), it was considered necessary to examine whether the two groups could be considered equivalent before implementing the intervention. Analyzing the data showed that the two groups did not start at the same level, with the control group performing significantly better. After the intervention, both teaching methods proved equally effective, with the same average performance. As the pretest showed that the two groups did not start at the same level, with the control group being superior and the posttest showing the same average performance, the average improvement of the two groups after the intervention was checked. The experimental group's mean improvement of 2.43 is statistically more significant than the control group's mean improvement, demonstrating the effectiveness of the ScratchJr programming environment in fostering CT in preschoolers. This finding was in line with the reviewed studies, indicating the efficacy of ScratchJr in fostering CT, and echoes the findings of other studies where boys and girls did not differ in their performance in understanding basic programming concepts [35,36], although boys slightly outperformed in this study.

Despite this, the children in the experimental group improved more than those in the control group, which confirms the effectiveness of the digital application ScratchJr. Regarding the age group, although both teaching methods led to the same mean performance at the post-test, the same was not true for the mean improvement, which was slightly higher for the five-year-old preschoolers than the four-year-old preschoolers. This finding is in the same direction as other studies' conclusions [34,35,45] that children show developmental differences in programming understanding, with younger (4–5) children achieving less than older (5–6) children. In addition, preschoolers in the experimental group showed a statistically more significant improvement in understanding basic programming concepts than the control group, reinforcing the view of the effectiveness of ScratchJr. In contrast, for 4-year-old preschoolers, both teaching methods proved to be equivalent, as the mean improvement of preschoolers was about the same.

Looking at the statistical data of the second research question, which powerful ideas of preschool CT can children develop through a short-term educational intervention using ScratchJr, both teaching methods can be considered equivalent in cultivating the basic computational concepts of modularity and control structures. On the posttest for the two powerful ideas mentioned above, the average performance of the two groups was about the same. The ScratchJr programming environment effectively promoted representation, algorithms and hardware/software programming concepts. In particular, the statistical analysis showed a significant difference in the mean performance between the experimental and control groups regarding the programming concepts of representation and hardware/software. The experimental group performed better than the control group.

**Fig. 6:**
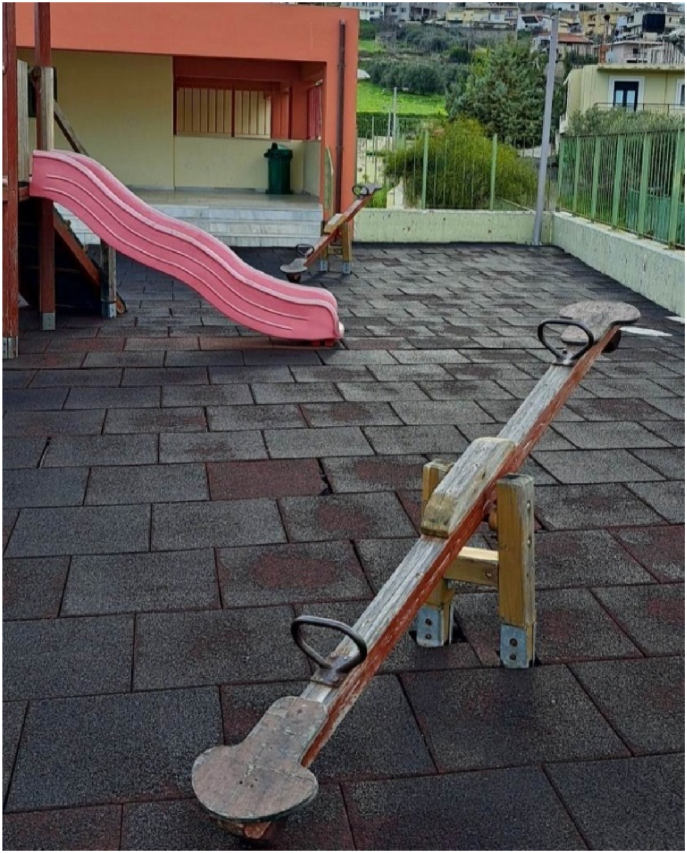
The school playground with the seesaws.

Regarding algorithms, the data analysis revealed a statistically more significant improvement in the experimental group. These results agree with Strawhacker et al. [34], who showed that young students developed CT skills by understanding the sequence and interpretation of symbols using ScratchJr. Finally, as the control group showed statistically more substantial performance than the experimental group, the conclusions from the data analysis regarding the understanding of the computational concept of debugging are impressive. Although previous studies have suggested that preschool children's engagement with ScratchJr can improve their skills in code error detection and correction [3,34], the findings in this study are inconsistent. The two questions included in the assessment tool that measure this concept are likely responsible for the control group's higher performance in understanding the concept of debugging. These questions present a picture of a seesaw that does not go up and down. The children are asked to circle the picture in which the seesaw works correctly. Simultaneously, two seesaws were placed in the schoolyard where the research was conducted for the young pupils to play during recess. Therefore, the children know the correct use of the seesaw through experience. Therefore, when asked the two questions measuring their understanding of debugging, the children in the control group chose the correct answers because they have an experiential knowledge of the correct way to operate the seesaw (Fig. 6).

Overall, ScratchJr engaged young learners. It was an enjoyable learning experience. At the end of one day's activities, they were excited about the next day's activities. After learning to use ScratchJr and completing the teaching intervention, they strongly wanted to create their projects, confirming that it is a programming environment that promotes expressing and creating [19]. The ScratchJr programming environment is a compelling and developmentally appropriate way to promote CT and coding skills at a preschool level.

Using the ScratchJr programming environment, the main finding of this research is that it is possible to develop CT in preschool education [3,[34], [35], [36],^43^,^45^]. While there are positive learning outcomes that result from designing activities that promote learning through the use of a screen, such as in the present research using the ScratchJr programming environment to cultivate CT, recent studies show that preschool children's screen use for more than one to 2 h has adverse effects on their development and behaviour [53,54]. In particular, distraction problems and language deficits are associated with children's screen use for more than 1 h. Simultaneously, children show weaknesses in social and emotional development [55]. The adoption of a pedagogical design based on learning through play, which seems to be reappearing in the scientific community as it promotes the child's multifaceted development [56], with the parallel integration of movement in the design of educational activities [57], seems to be the appropriate solution to reduce the negative consequences caused by children's exposure to screens, but also to take advantage of the positive effects that result from it. This could be achieved by including unplugged activities in developing CT using ScratchJr in kindergarten.

In addition, several studies have supported that unplugged activities and coding activities are the most appropriate ways to enhance CT in preschool children [58]. Unplugged activities allow for a more visual experience of CT and provide young learners with the necessary vocabulary for the subsequent coding activity, which will be carried out using a digital device [59]. Therefore, by moving from unplugged to plugged learning, preschool children can demonstrate their ability to understand basic programming concepts [35].

Some recommendations on teaching practices could be made to help develop CT skills in preschool children. Firstly, digital tools and multimedia files were attractive teaching tools that helped transform academic knowledge into school knowledge. Specifically, the psychological preparation activity, which included a multimedia file and introduced children to the teaching of the day's powerful idea, aroused children's interest and increased motivation to learn. Furthermore, participatory, collaborative techniques, such as workgroups and feedback discussions to cultivate metacognitive skills, fostered the active participation of all children. Teamwork allowed mutual assistance of its members and the correction of mistakes in a pleasant and supportive atmosphere. Cooperative learning matched the children's need for action and energy. Finally, it is deemed necessary to implement training programs for educators that will help them include plugged and unplugged activities in the educational process that will encourage computational thinking in kindergarten.

### 3.3 Research limitations and suggestions for future studies

Although the results are considered positive, there were limitations in the research design, which does not allow the generalization of the results. Firstly, the study sample was small and came from a particular area of Heraklion Prefecture. Future studies could increase the sample size and use random sampling for a more representative sample. This will also necessitate the utilization of more rigorous statistical tests. For example, we acknowledge that the paper lacks comparisons involving multiple variables and their interactions. Additionally, it does not identify latent variables that congregate into groups (factors), nor does it account for combinations of variables when predicting discrete outcome variables. Secondly, assessment tools, including multiple-choice questions, were used. As the powerful idea of the design process is, an unrestricted and continuous process was excluded from the measurements that may affect the analysis of CT skills. Future studies could use appropriate assessment tools, such as observation and open-ended questionnaires, to include the design process in their measurements. Thirdly, the reliability of the self-designed instruments might need to be stronger. To evaluate ScratchJr activities, the researcher created an identical questionnaire with multiple-choice questions similar to the TechCkeck instrument. However, the self-designed assessment tool may introduce a potential measurement bias. A future study could eliminate this limitation. Fourth, a prospective inquiry characterized by a judiciously constructed sample could perform a cross-comparative analysis of children's age and gender. Another limitation of this study is that, although we made substantial efforts to ensure the questionnaires' uniformity, there exists a potential for greater standardization. For example, Question 1 pertains to matters of weight, while Question 2 pertains to experiences derived from learners' priori knowledge. This analytic search seeks to discern distinct performance differences, particularly concerning cognitive abilities, between male participants at age five and their female counterparts at age four. Finally, future studies could include parental involvement in the design of the educational intervention with take-home activities. Research indicates parental involvement positively impacts students' CT skills [60].

## 4 Conclusion

Main Findings: The study demonstrated that the ScratchJr programming environment significantly enhances computational thinking (CT) and basic programming concepts in preschool children aged 4–6 years through a three-week educational intervention.

Significance: This research underscores the effectiveness of ScratchJr in early childhood education, highlighting its potential to equip young learners with foundational problem-solving skills necessary for the modern world.

Implications: The positive outcomes suggest that integrating developmentally appropriate programming environments like ScratchJr into preschool curricula can foster essential CT skills, preparing children for future academic and professional challenges.

## Data availability

Data is available upon request.

## Ethical approval statement

The Research Ethics Committee of the University of Crete approved the research, with approval number 5454/261, dated March 23, 2022.

## Funding

This research did not receive any specific funding.

## CRediT authorship contribution statement

**Papadakis Stamatios:** Writing – review & editing, Writing – original draft, Visualization, Validation, Software, Project administration, Methodology, Conceptualization. **Louka Konstantina:** Resources, Investigation, Data curation, Conceptualization.

## Declaration of competing interest

The authors declare the following financial interests/personal relationships which may be considered as potential competing interests:

Stamatios Papadakis reports a relationship with Heliyon that includes: board membership. If there are other authors, they declare that they have no known competing financial interests or personal relationships that could have appeared to influence the work reported in this paper.

## Figures and Tables

**Figure:**
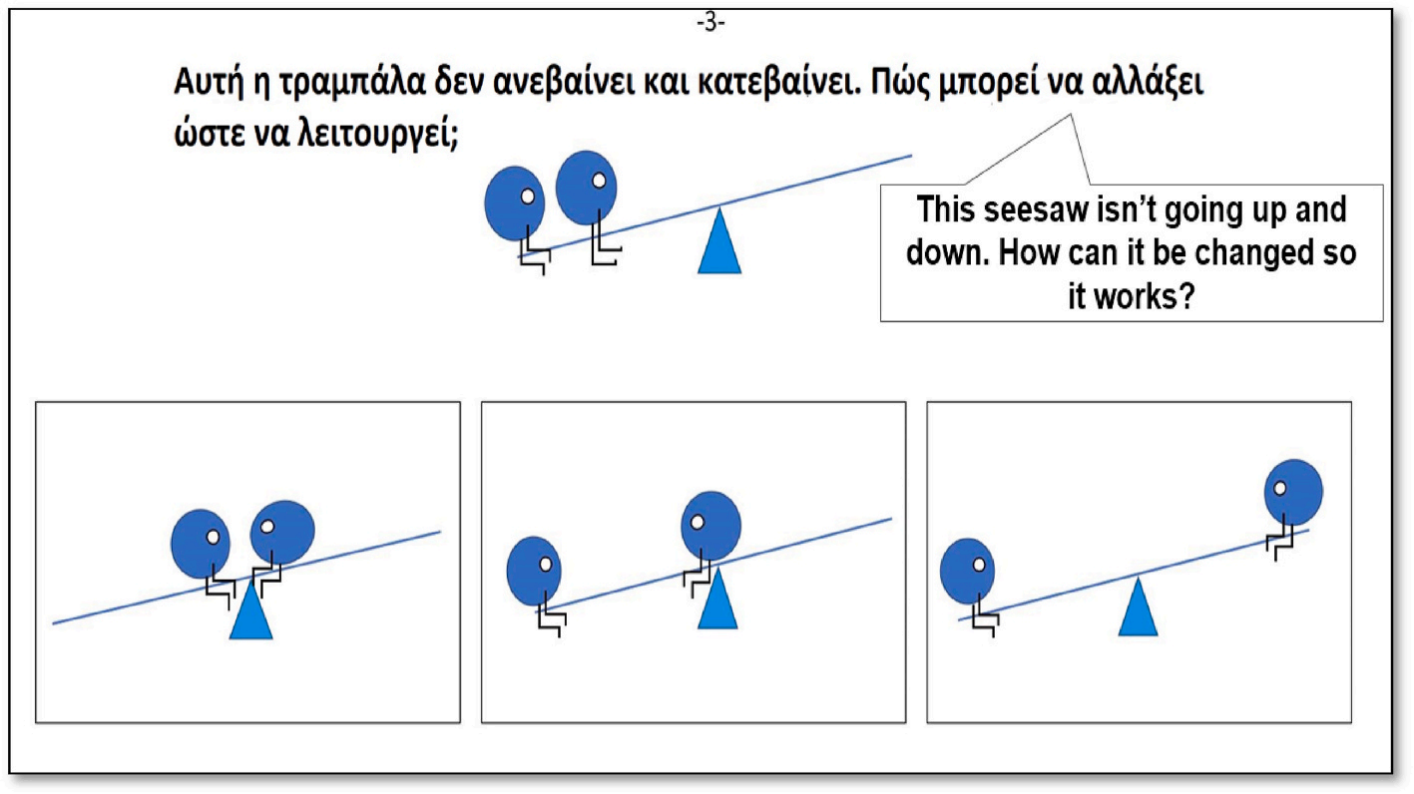


**Figure:**
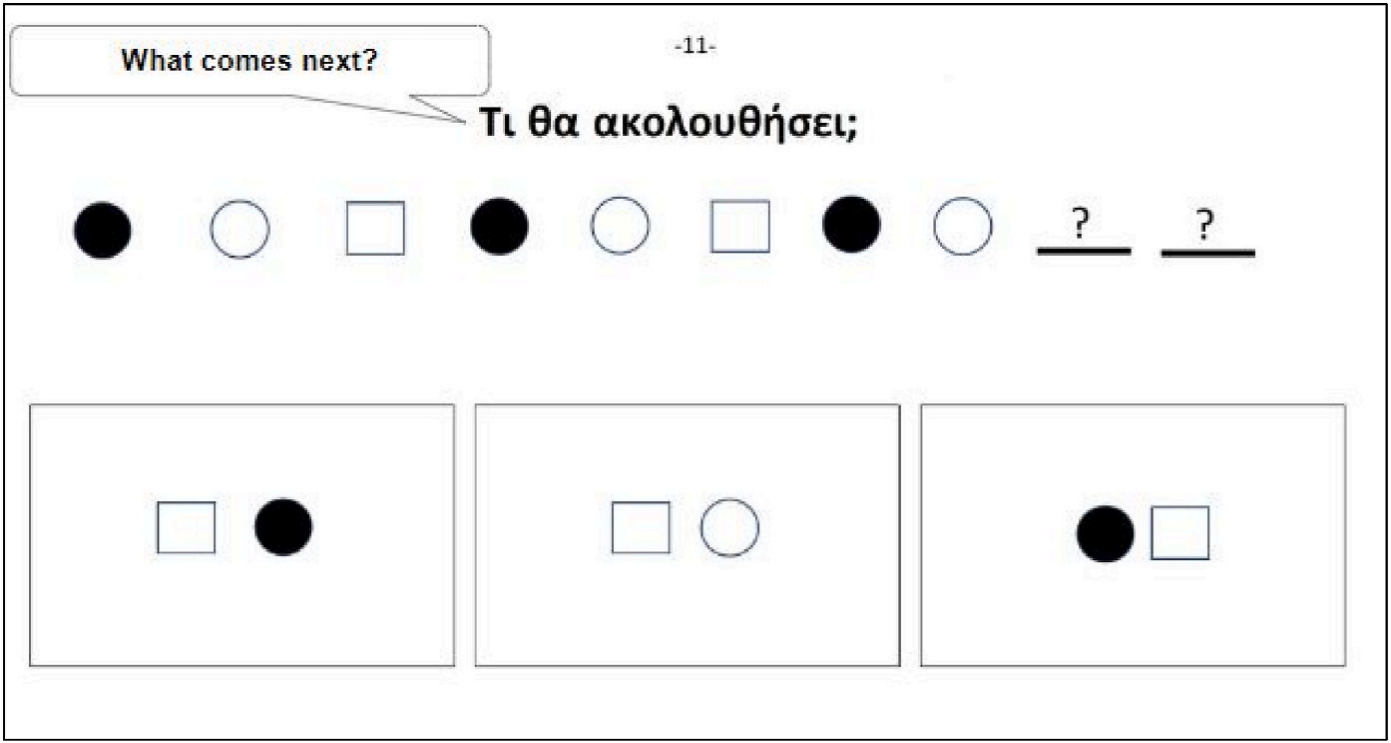


**Figure:**
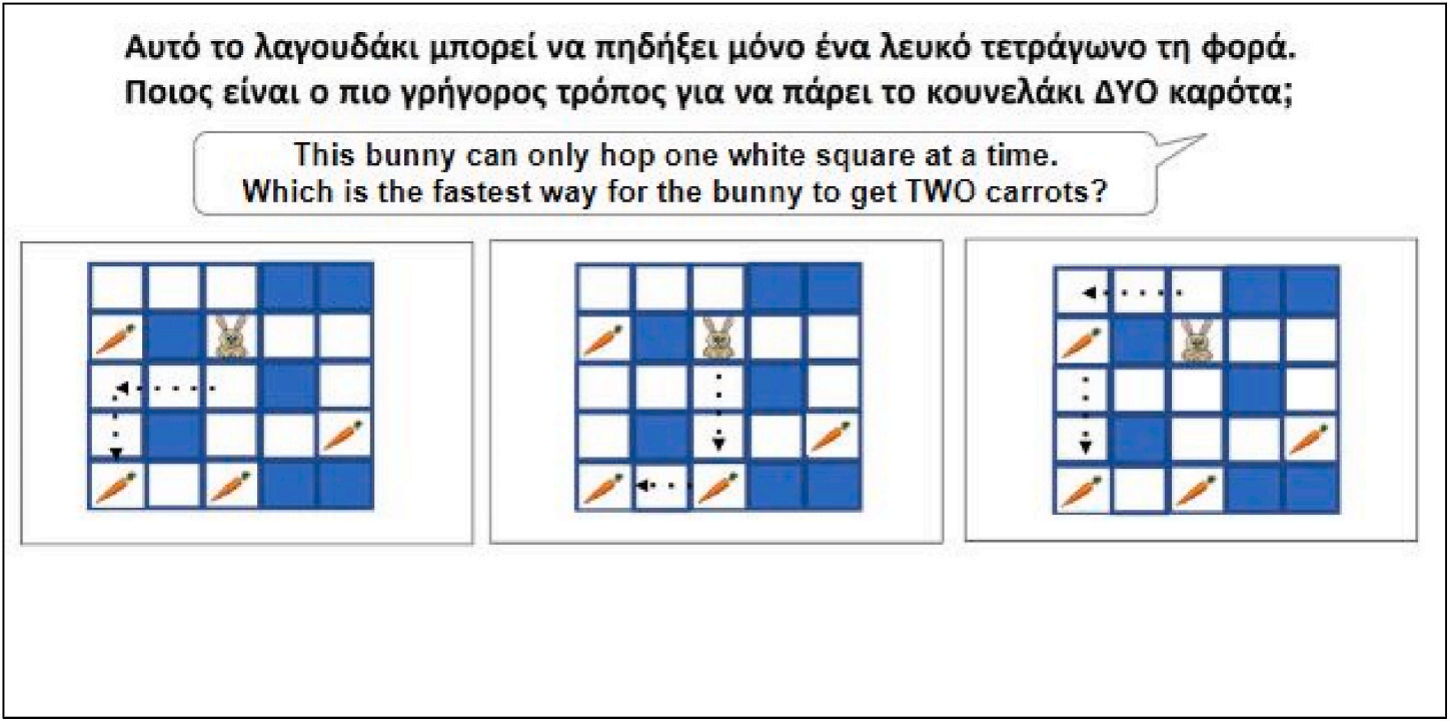


**Figure:**
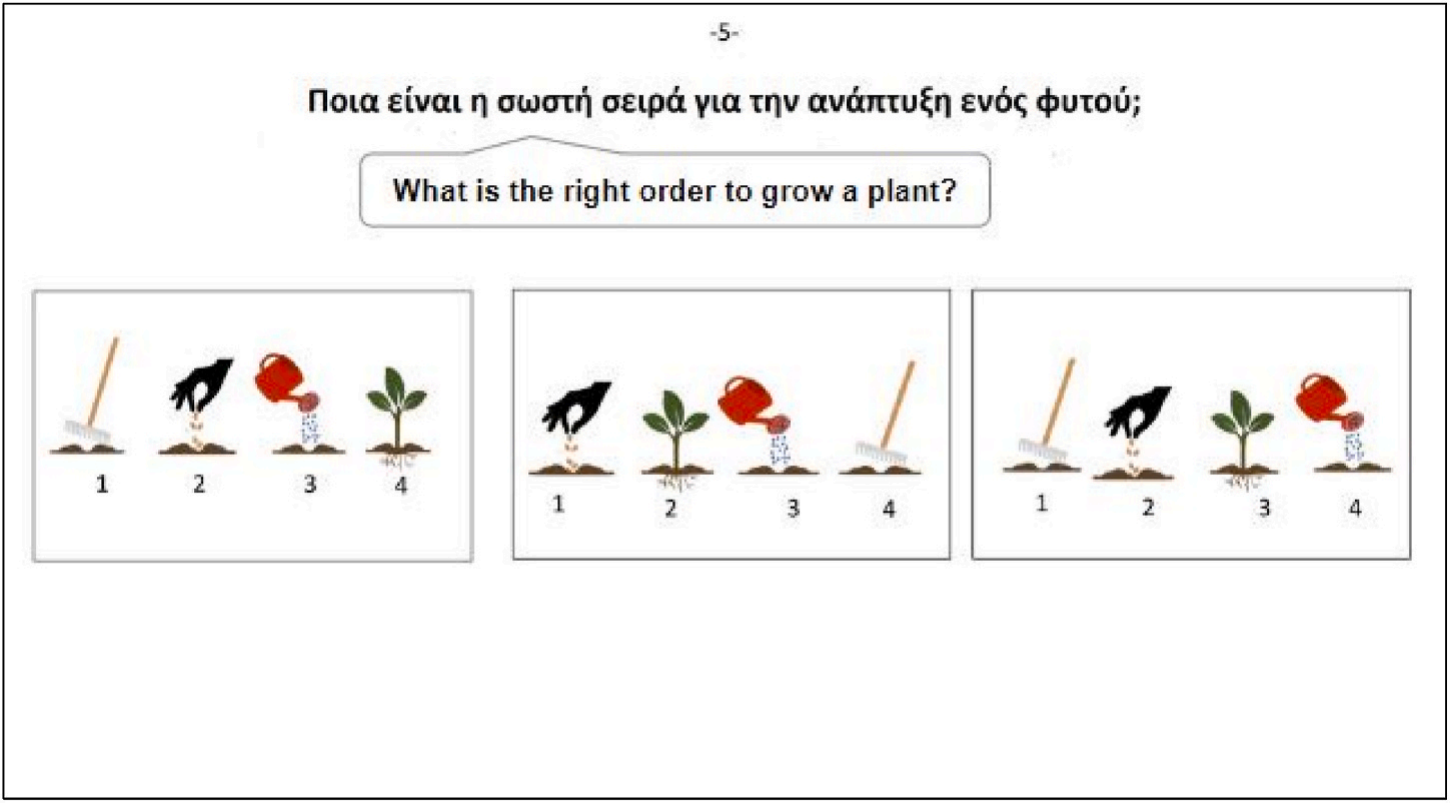


**Figure:**
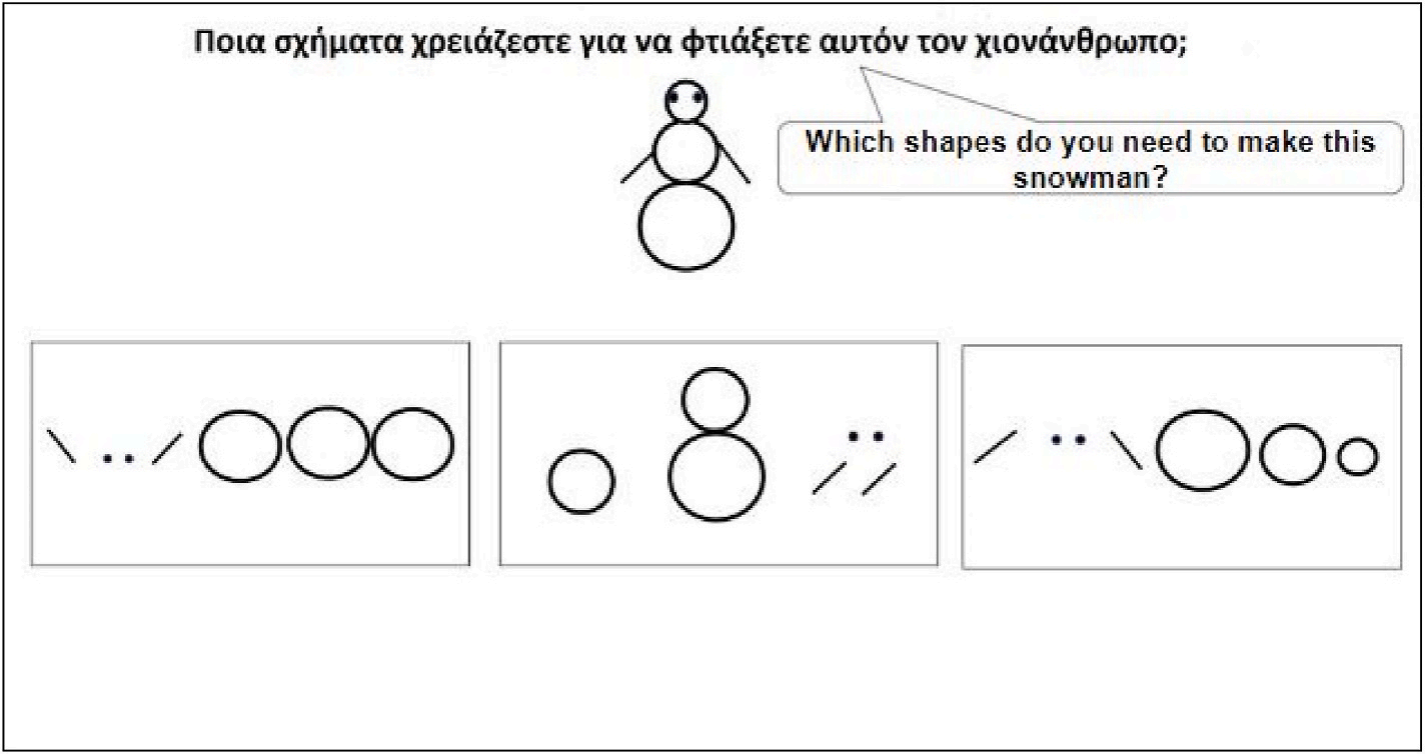


**Figure:**
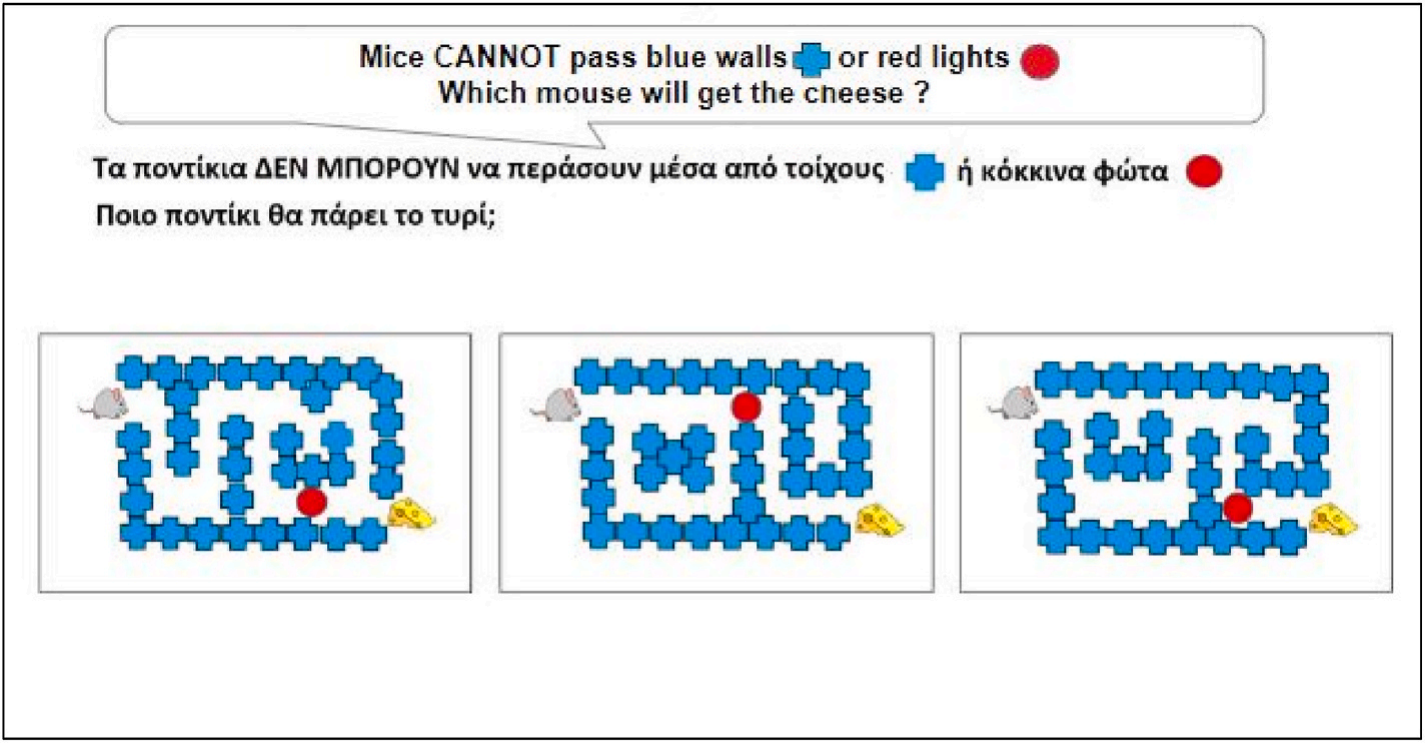


**Figure:**
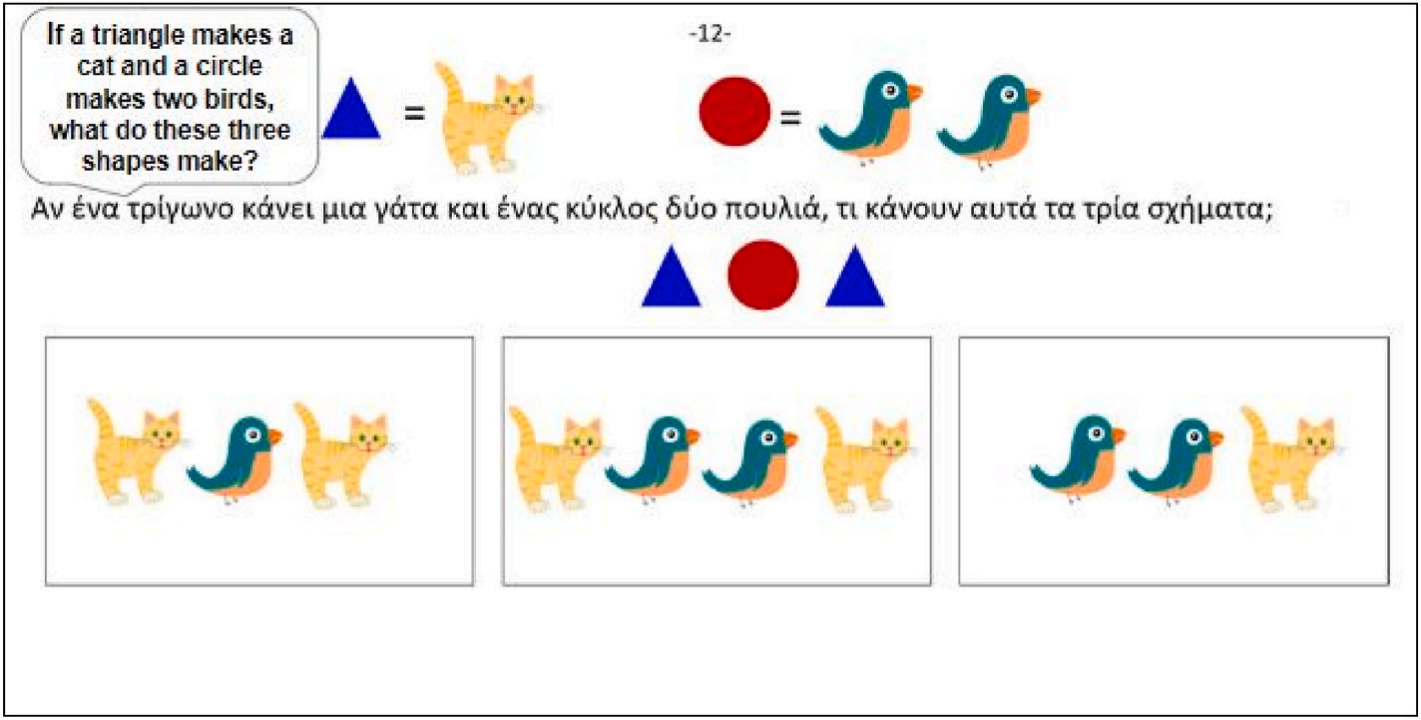


**Figure:**
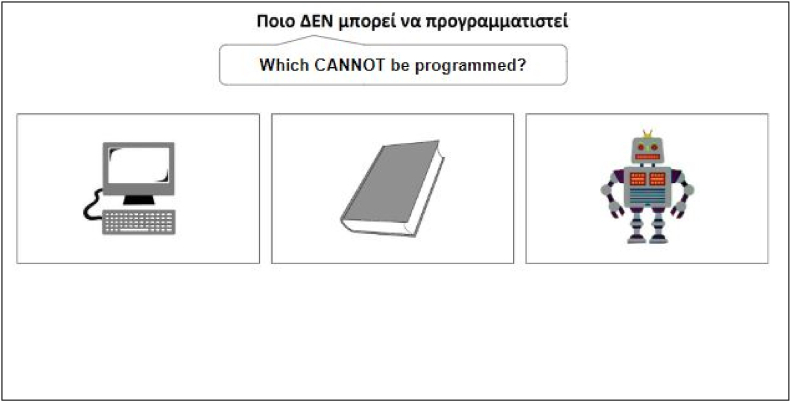


**Figure:**
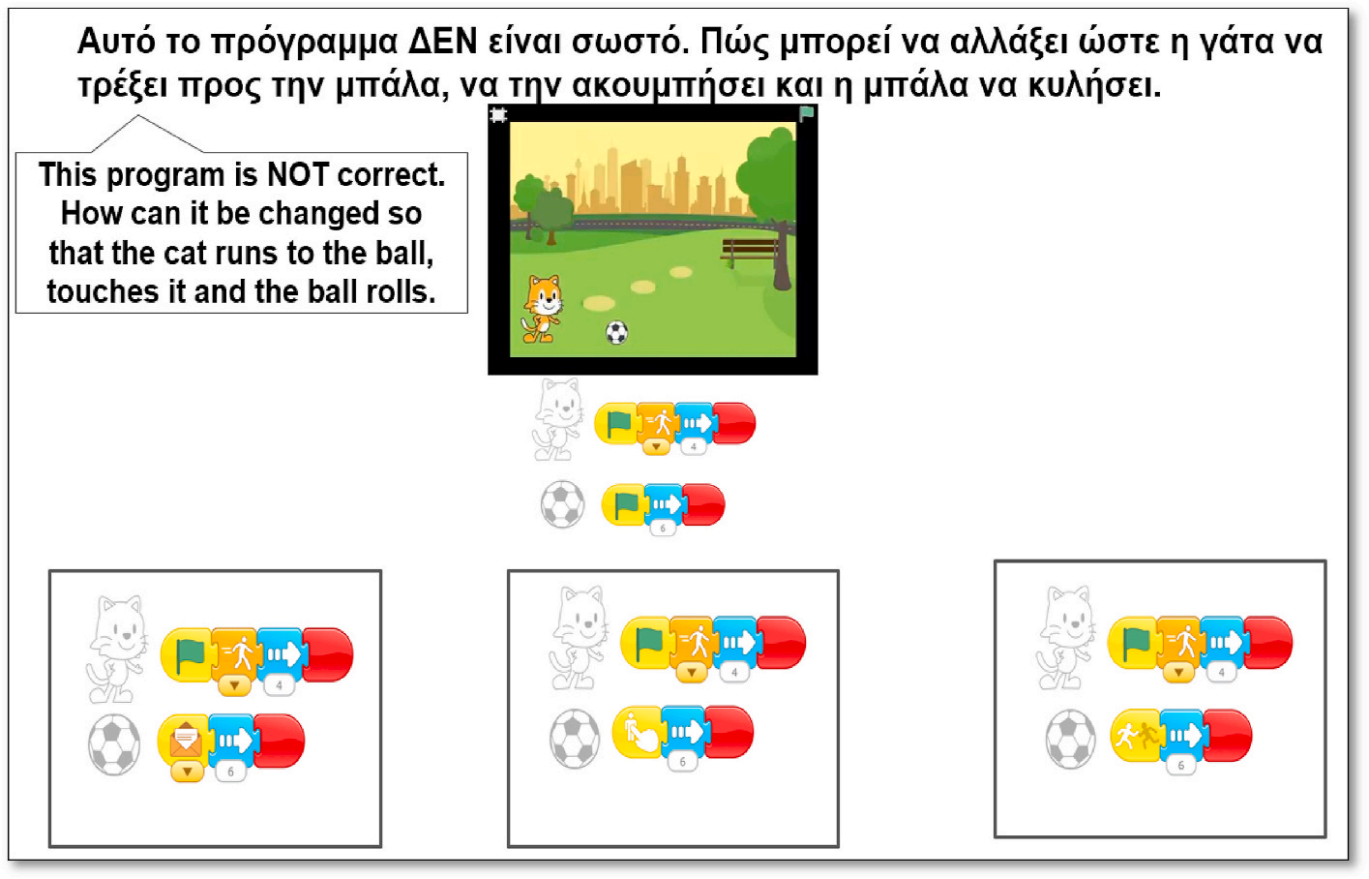


**Figure:**
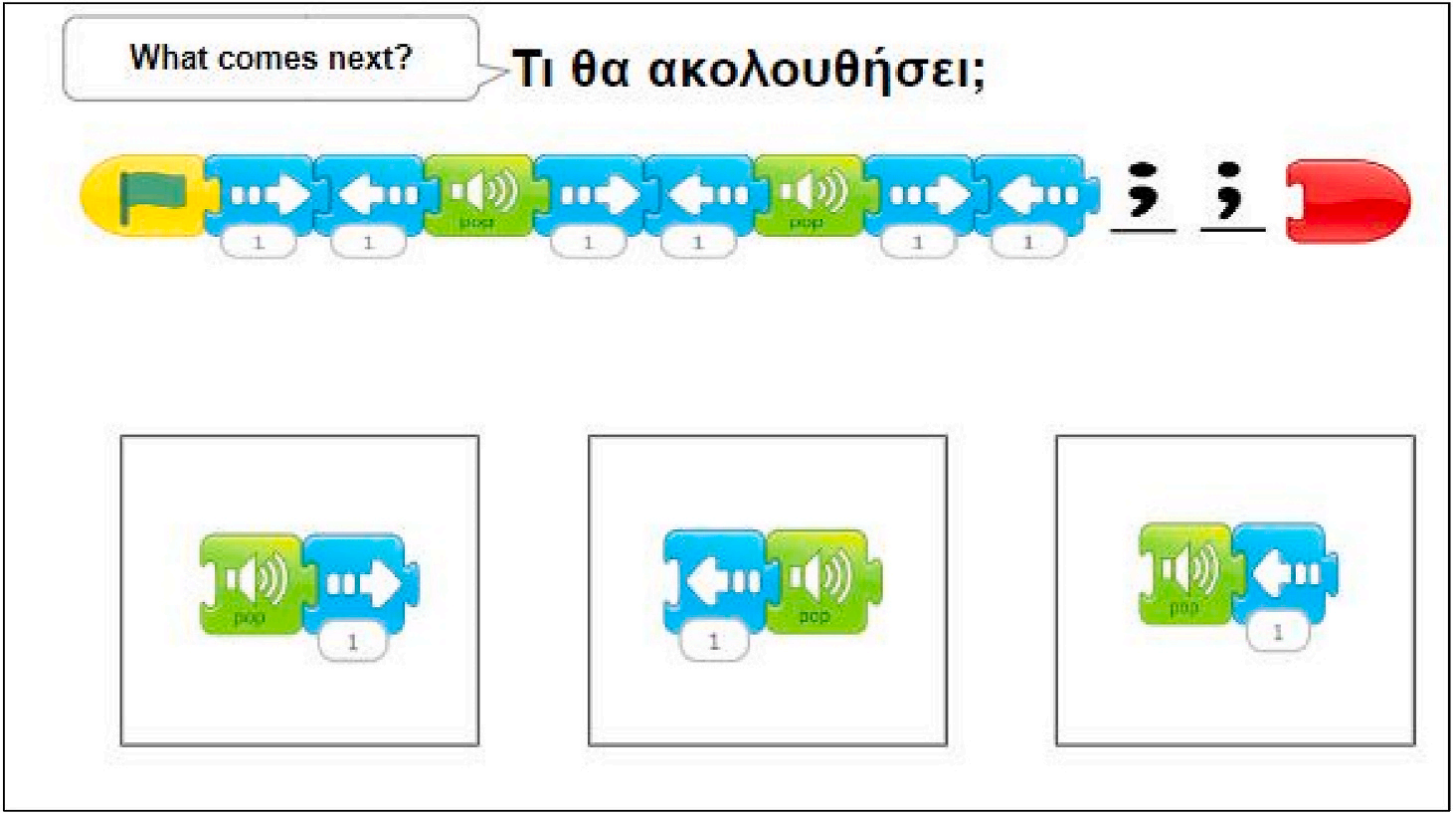


**Figure:**
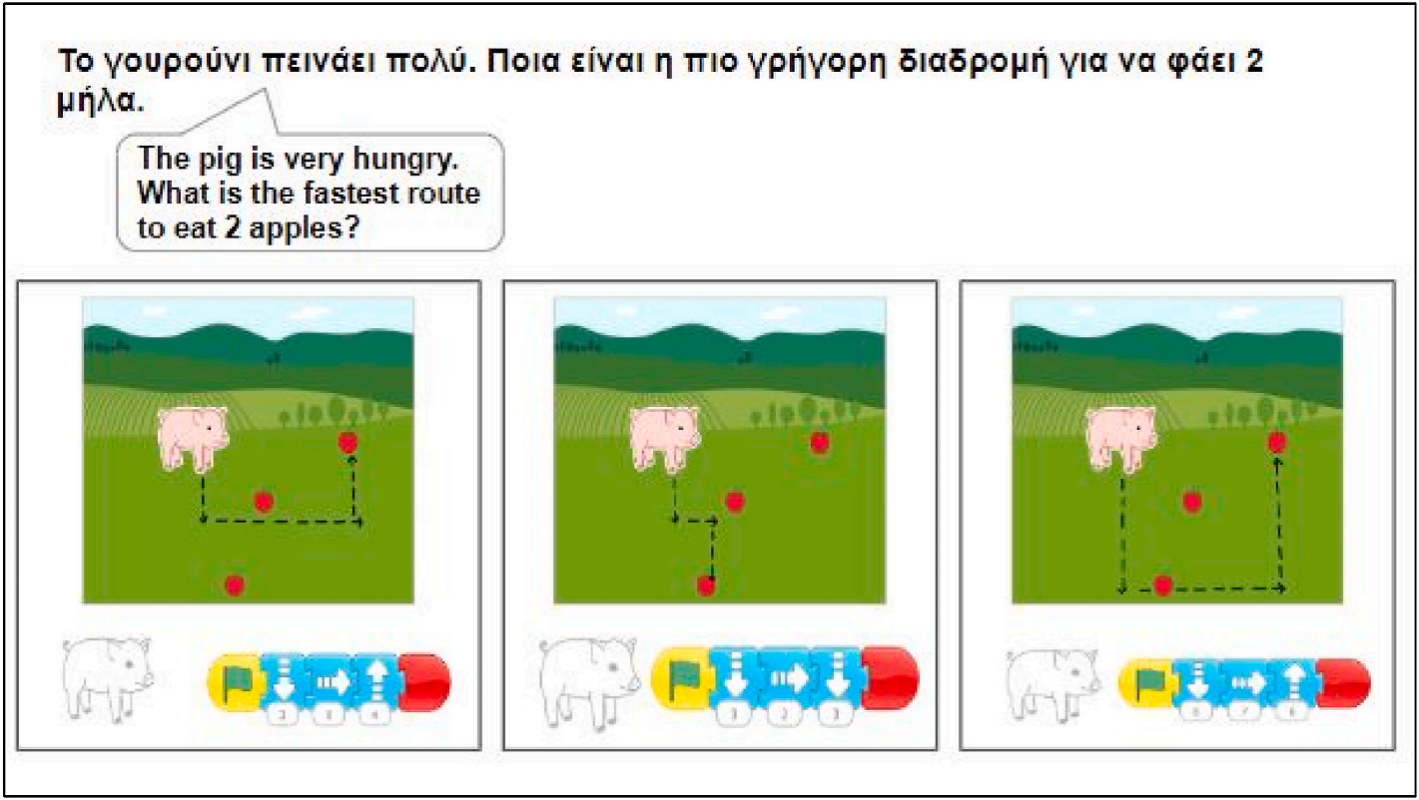


**Figure:**
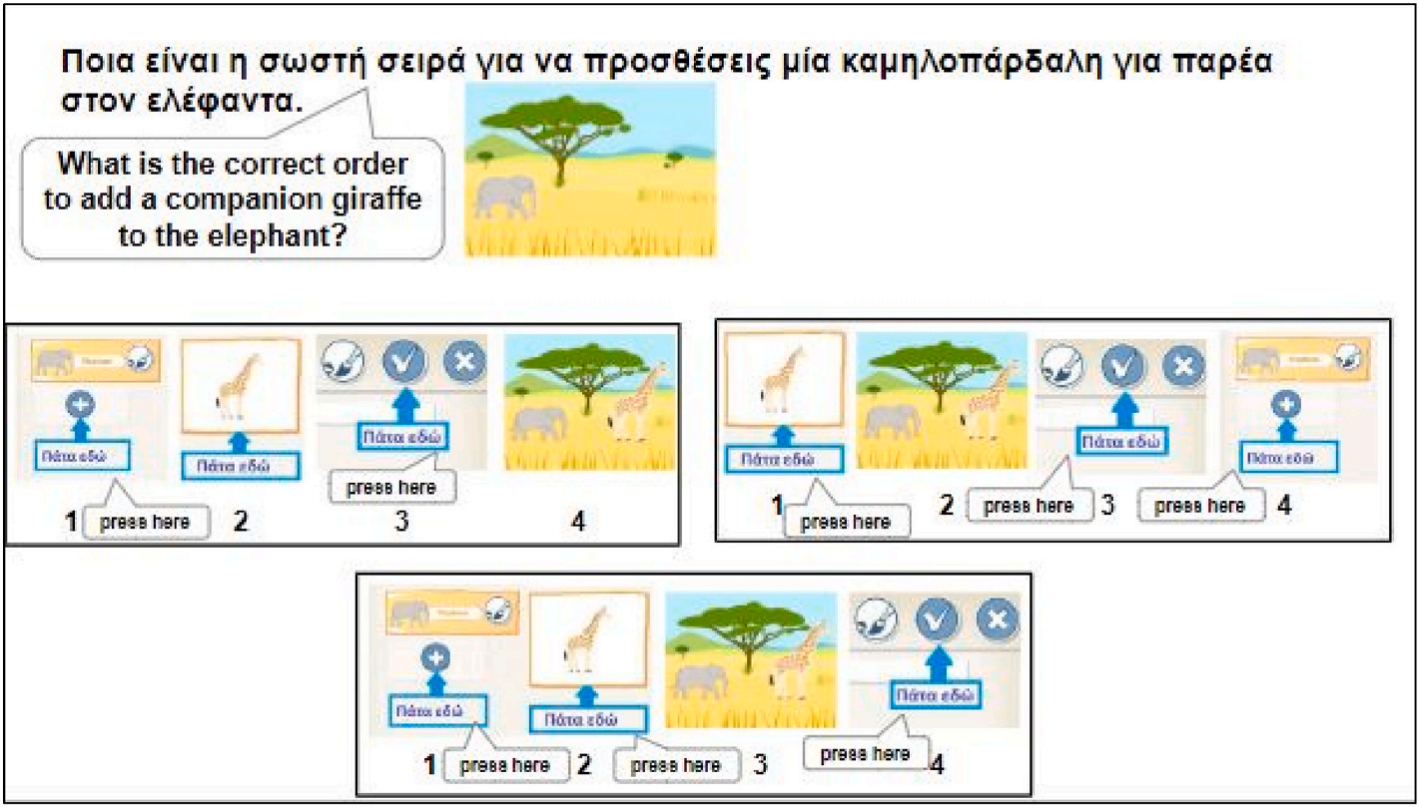


**Figure:**
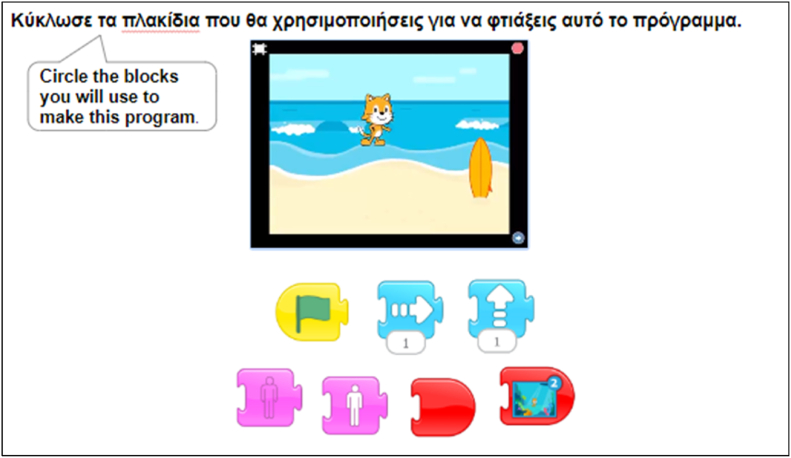


**Figure:**
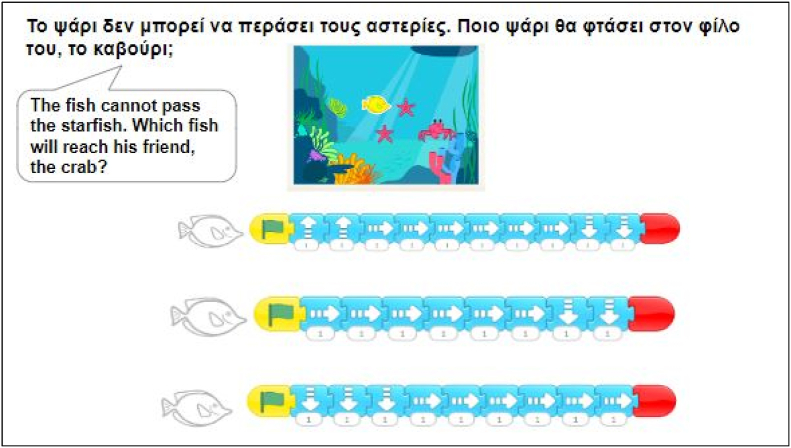


**Figure:**
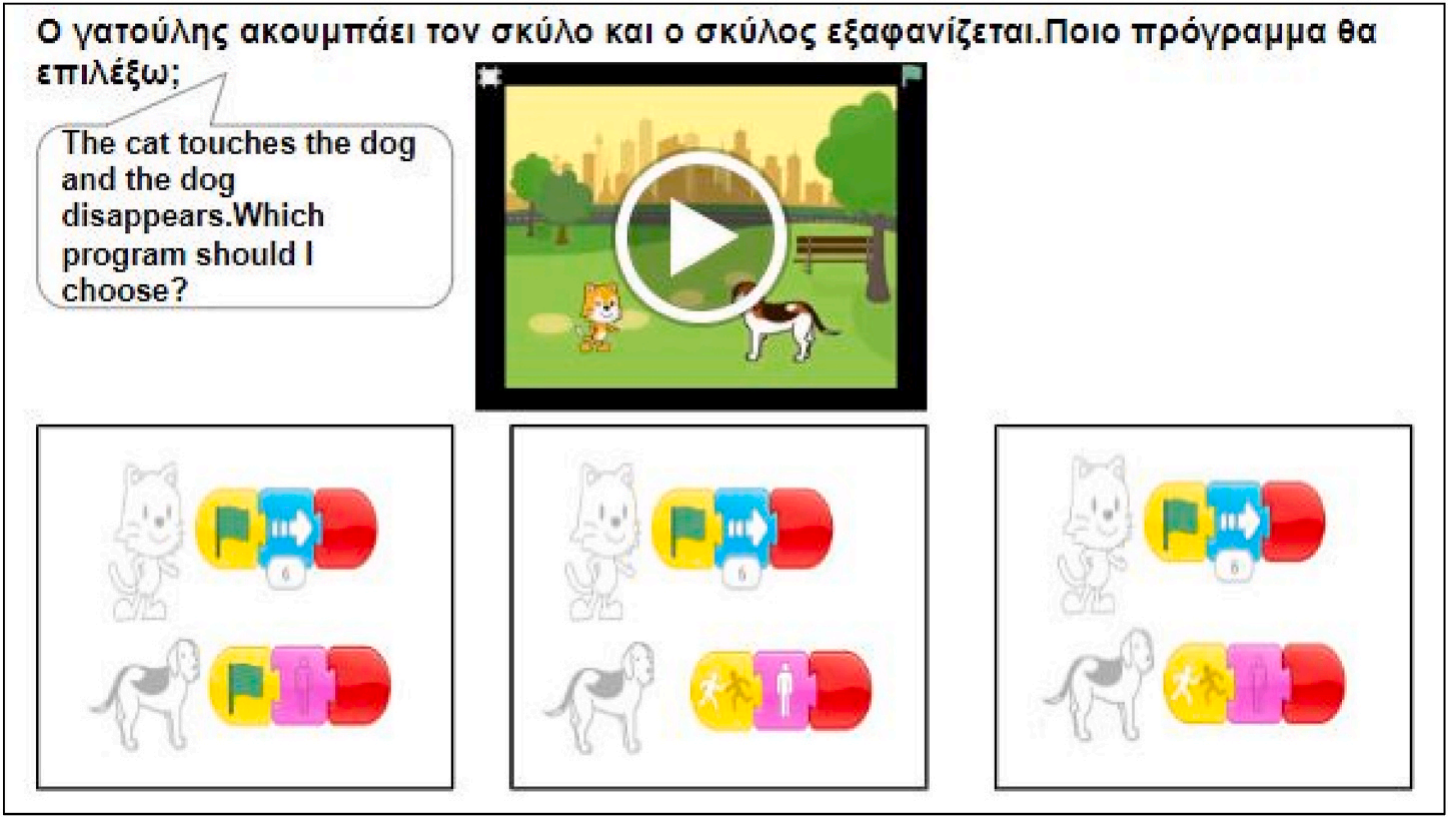


**Figure:**
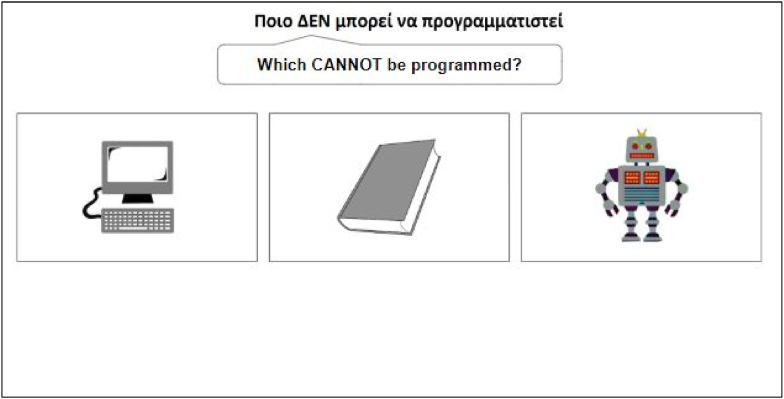


## Appendix 1

TechCheck-K assessment tool example pages.

CT domain: Debugging.

CT domain: Algorithms.

Task type: missing symbol series.

CT domain: Algorithms.

Task type: shortest path puzzles.

CT domain: Algorithms.

Task type: sequencing challenge.

CT domain: Modularity.

Task type: object decomposition.

CT domain: Control Structures.

Task type: obstacle maze.

CT domain: Representation.

Task type: symbol shape puzzle.

CT domain: Hardware/software.

Task type: Identifying technological concepts.

## Appendix 2

Researcher created assessment tool example pages.

CT domain: Debugging.

CT domain: Algorithms.

Task type: missing symbol series.

CT domain: Algorithms.

Task type: shortest path puzzles.

CT domain: Algorithms.

Task type: sequencing challenge.

CT domain: Modularity.

Task type: object decomposition.

CT domain: Control Structures.

Task type: obstacle maze.

CT domain: Representation.

Task type: symbol shape puzzle.

CT domain: Hardware/software.

Task type: Identifying technological concepts.

## Footnotes

Supplementary data to this article can be found online at https://doi.org/10.1016/j.heliyon.2024.e30482.
