# Supplementary material for: Enhancing computational thinking in early childhood education through ScratchJr integration
Source: Heliyon. 2024 Apr 30;10(10):e30482. doi: 10.1016/j.heliyon.2024.e30482 (PMC11109739; doi:10.1016/j.heliyon.2024.e30482)

**Ποιο ΔΕΝ μπορεί να προγραμματιστεί**

**Which CANNOT be programmed?**

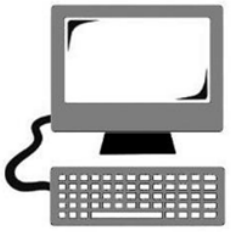

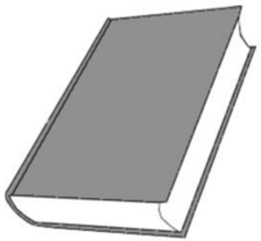

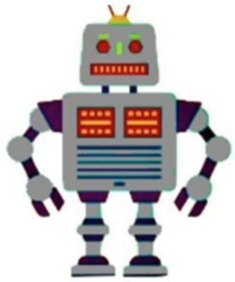

**Ποιο λειτουργεί σαν ένας υπολογιστής.**

**Which works the most like a computer?**

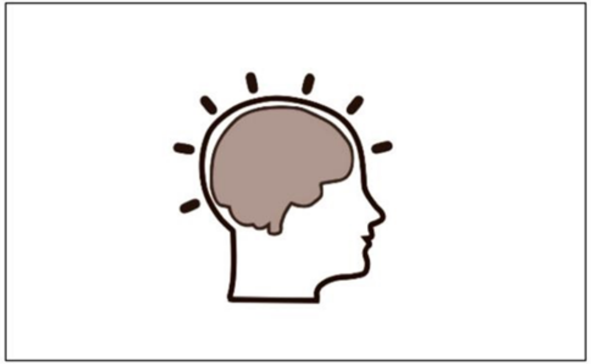

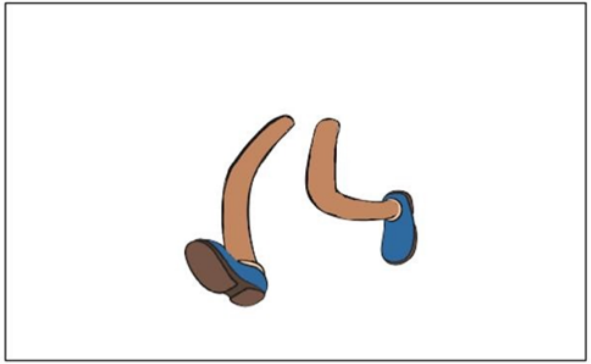

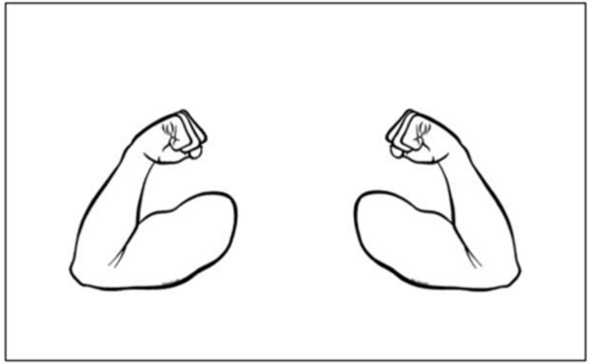

**Αυτή η τραμπάλα δεν ανεβαίνει και κατεβαίνει. Πώς μπορεί να αλλάξει ώστε να λειτουργεί;**

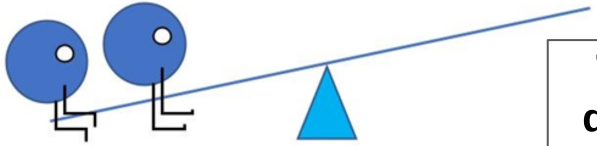

This seesaw isn't going up and down. How can it be changed so it works?

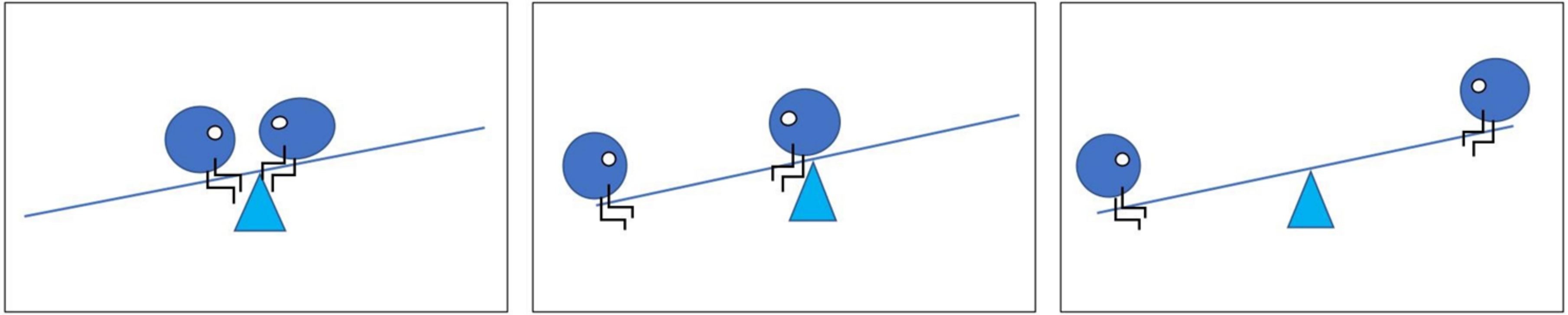

**Αυτή η τραμπάλα δεν ανεβαίνει και κατεβαίνει. Πώς μπορεί να αλλάξει ώστε να λειτουργεί;**

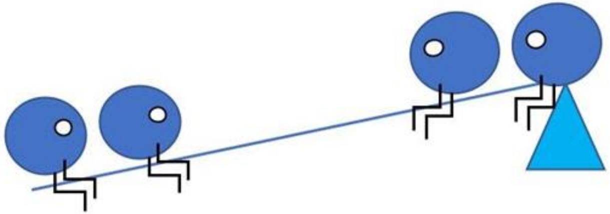

**This seesaw isn’t going up and down. How can it be changed so it works?**

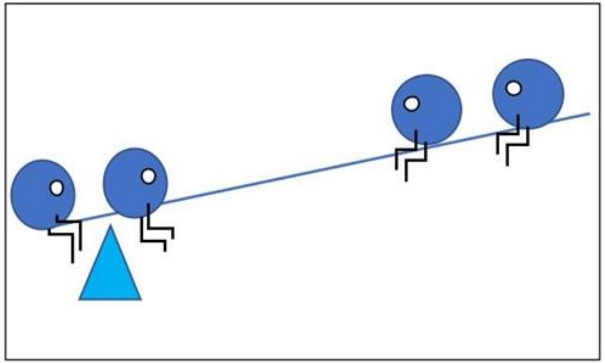

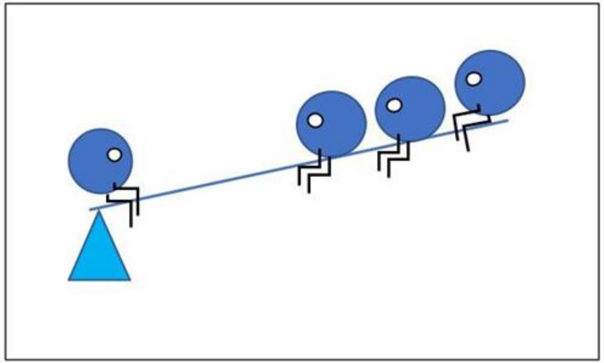

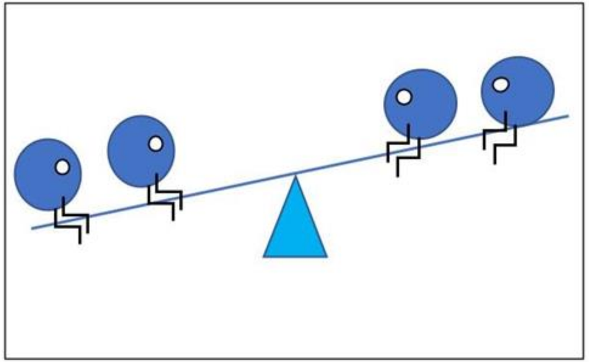

## Ποια είναι η σωστή σειρά για την ανάπτυξη ενός φυτού;

**What is the right order to grow a plant?**

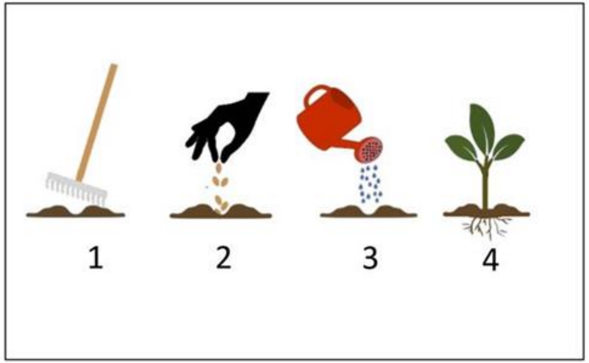


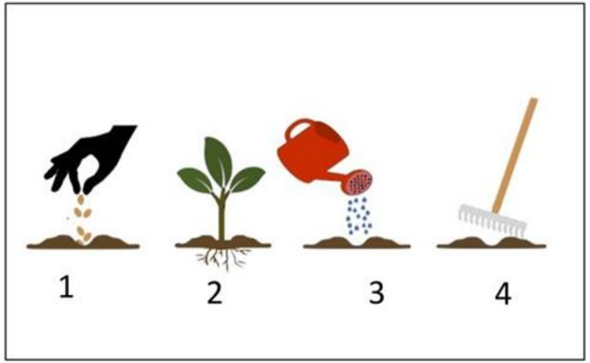


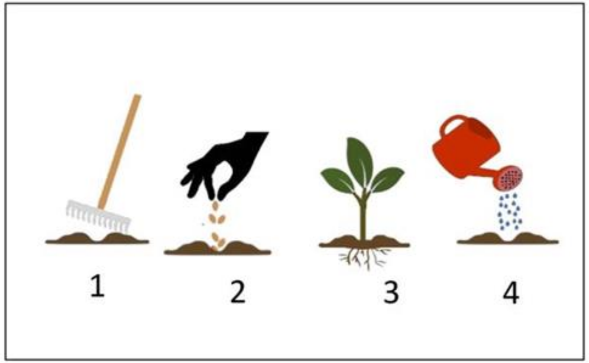

**Ποια σχήματα μπορείτε να χρησιμοποιήσετε για να το φτιάξετε;**

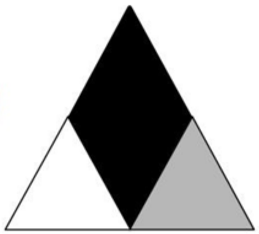

**Which shapes can you use to make this?**

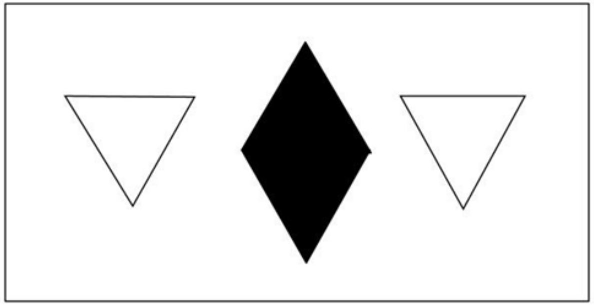

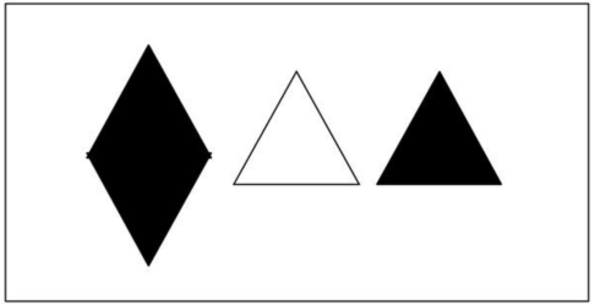

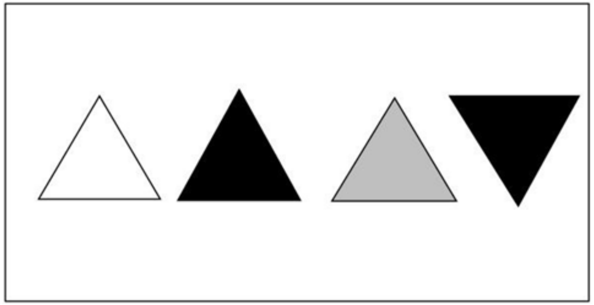

**Ποια σχήματα χρειάζεστε για να φτιάξετε αυτόν τον χιονάνθρωπο;**

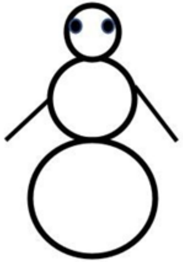

**Which shapes do you need to make this snowman?**

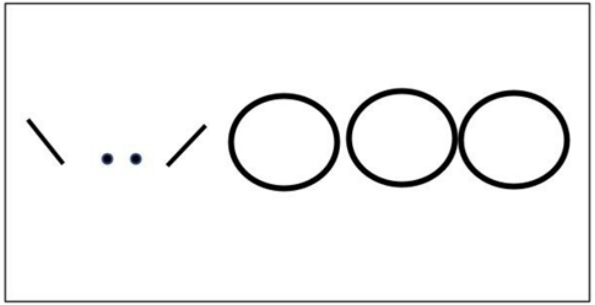

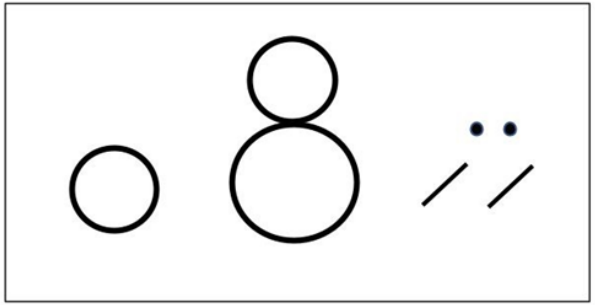

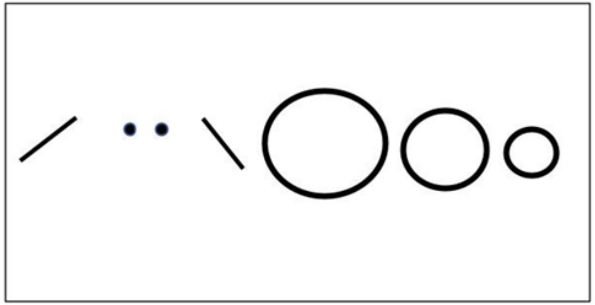

**Το λαγουδάκι μπορεί να πηδήξει μόνο ένα λευκό τετράγωνο τη φορά.**
**Ποιος είναι ο πιο γρήγορος τρόπος για να πάρει το κουνελάκι ΕΝΑ καρότο;**

**The bunny can only hop one white square at a time.**
**What is the fastest way for the bunny to get ONE carrot?**

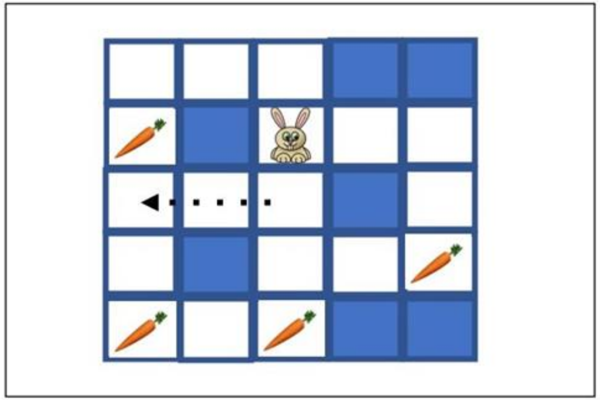

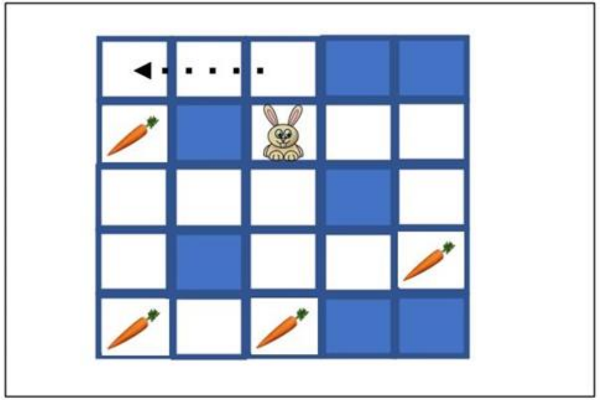

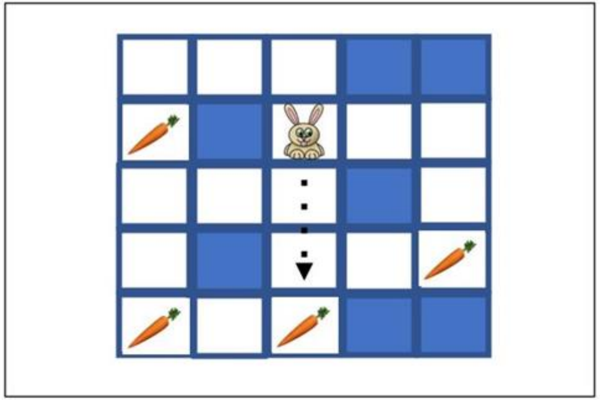

**Αυτό το λαγουδάκι μπορεί να πηδήξει μόνο ένα λευκό τετράγωνο τη φορά.**
**Ποιος είναι ο πιο γρήγορος τρόπος για να πάρει το κουνελάκι ΔΥΟ καρότα;**

**This bunny can only hop one white square at a time.**
**Which is the fastest way for the bunny to get TWO carrots?**

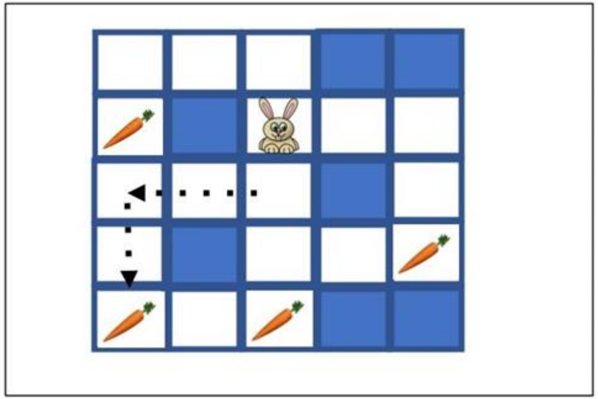

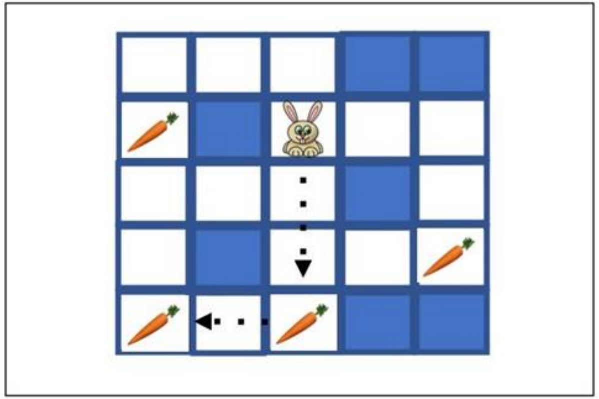

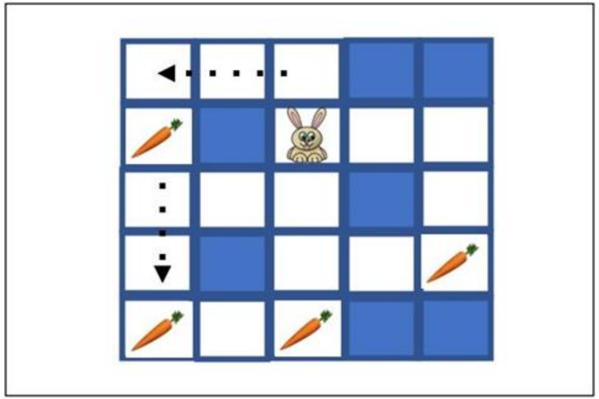

**What comes next?**

**Τι θα ακολουθήσει;**

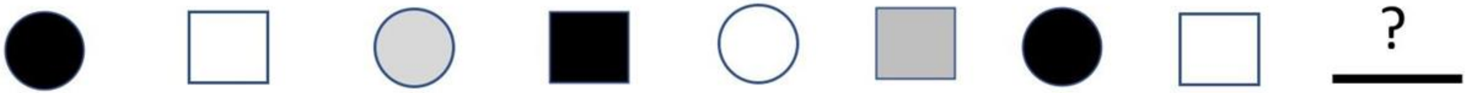


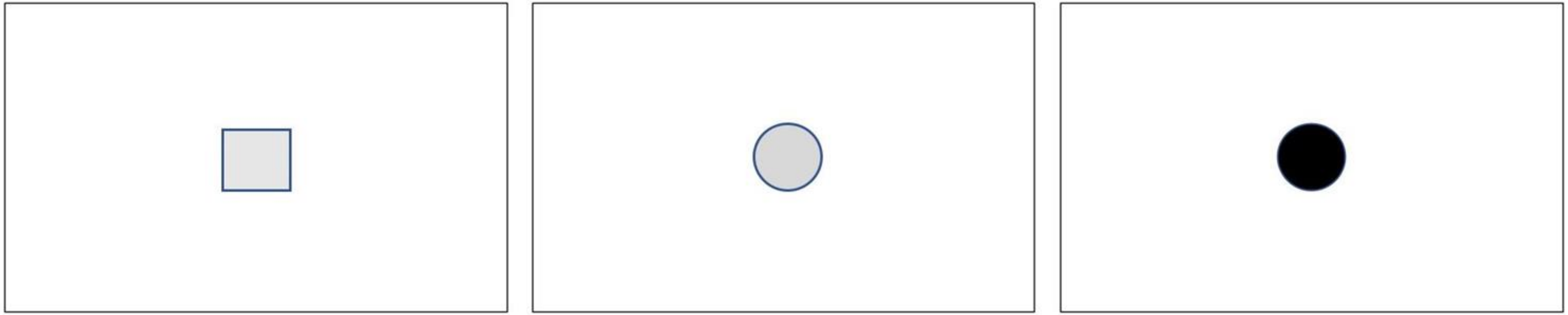

**What comes next?**

# Τι θα ακολουθήσει;

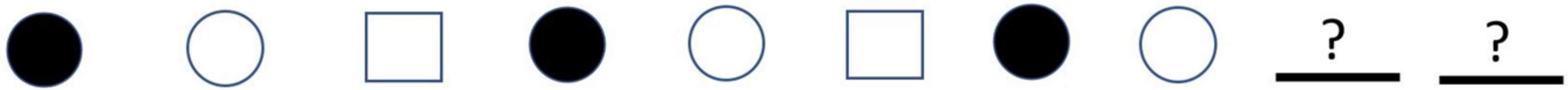


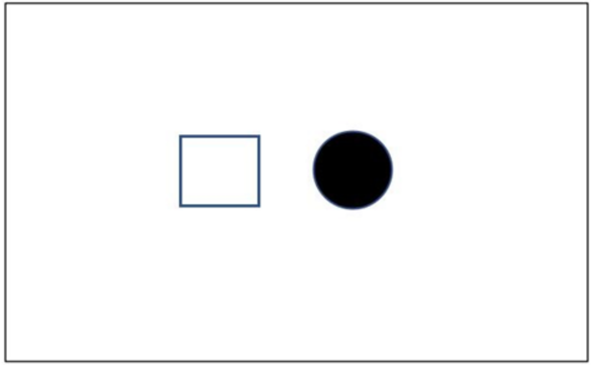

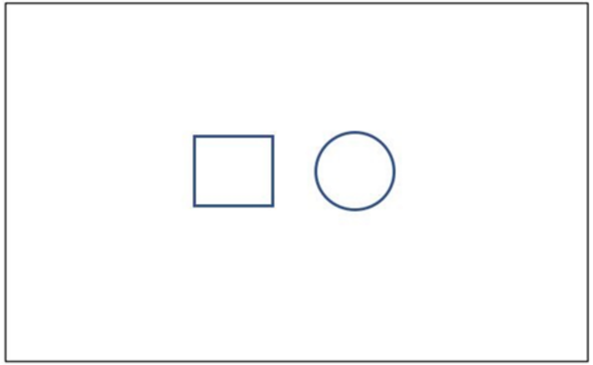

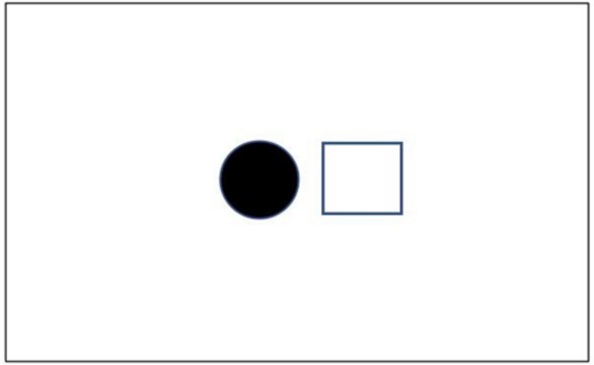

**If a triangle makes a cat and a circle makes two birds, what do these three shapes make?**

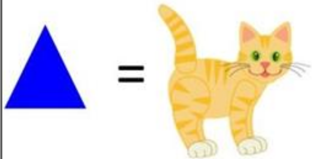

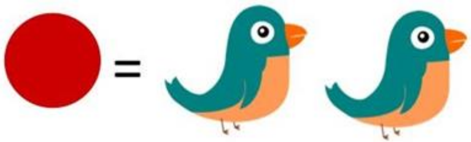

Αν ένα τρίγωνο κάνει μια γάτα και ένας κύκλος δύο πουλιά, τι κάνουν αυτά τα τρία σχήματα;

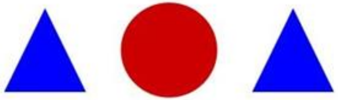

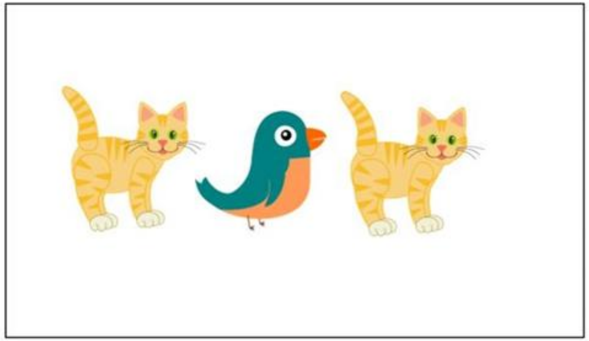

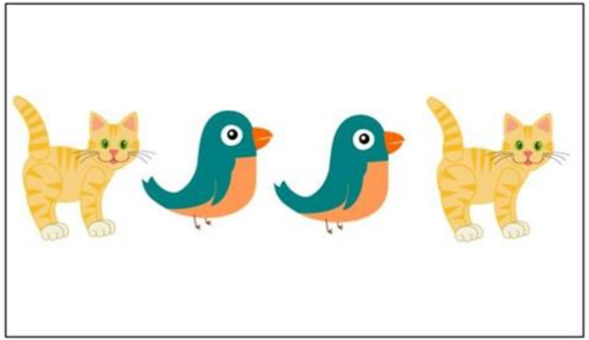

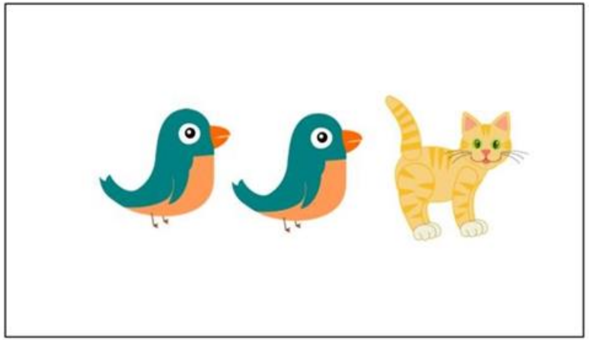

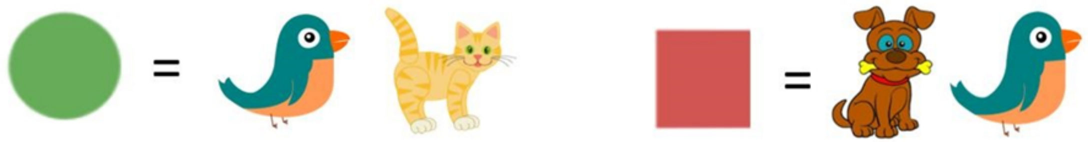

Ένας κύκλος κάνει ένα πουλί και μια γάτα. Ένα τετράγωνο κάνει έναν σκύλο και ένα πουλί; Τι κάνουν τα παρακάτω;

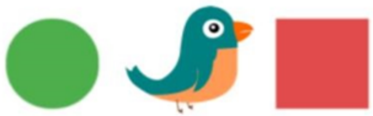

A circle makes a bird and a cat. A square makes a dog and a bird
What do these make?

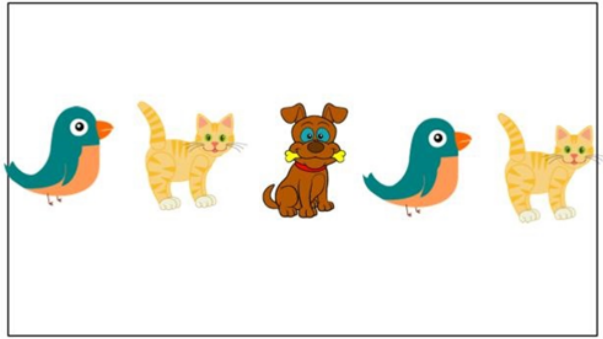

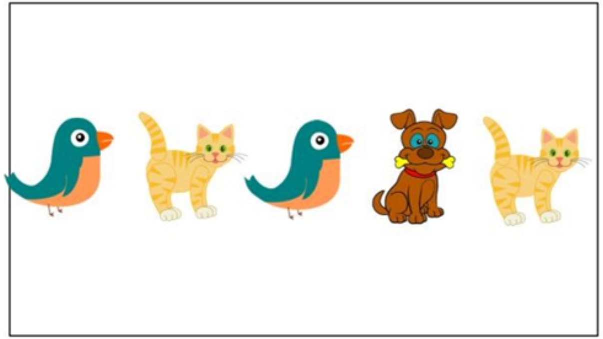

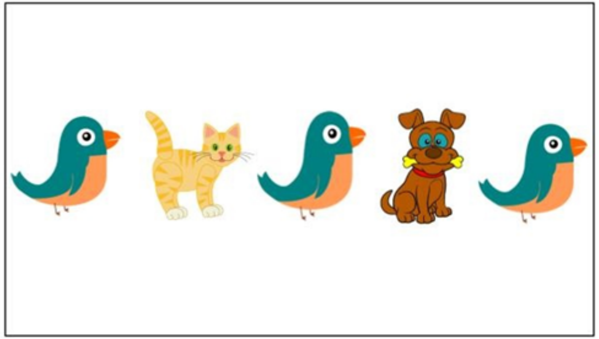

**Mice CANNOT pass blue walls or red lights**
**Which mouse will get the cheese ?**

**Τα ποντίκια ΔΕΝ ΜΠΟΡΟΥΝ να περάσουν μέσα από τοίχους ή κόκκινα φώτα**

**Ποιο ποντίκι θα πάρει το τυρί;**

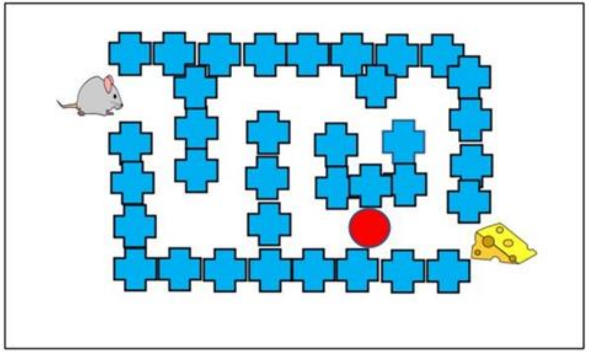

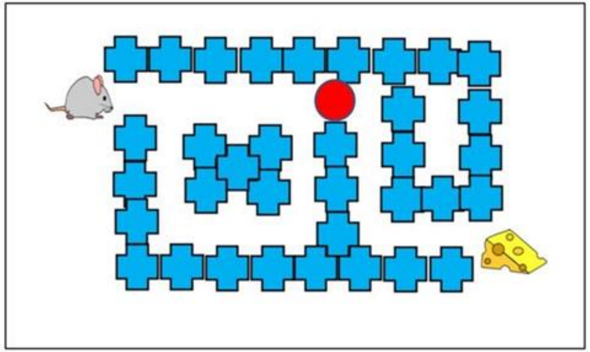

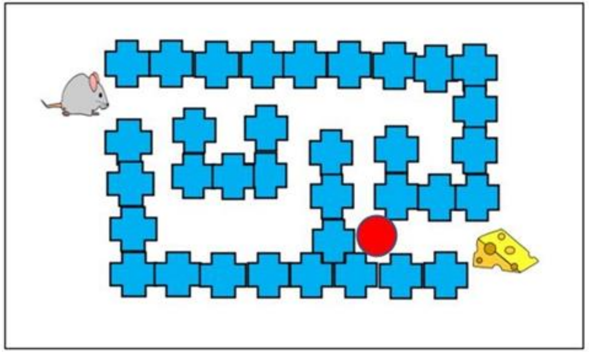

Τα ποντίκια ΔΕΝ ΜΠΟΡΟΥΝ να περάσουν μπλε τοίχους ή κόκκινα φώτα αλλά ΜΠΟΡΟΥΝ να περάσουν μαύρα τούνελ . Ποιο ποντίκι θα πάρει το τυρί;

**Mice CANNOT pass blue walls or red lights but CAN pass black tunnels**
**Which mouse will get the cheese?**

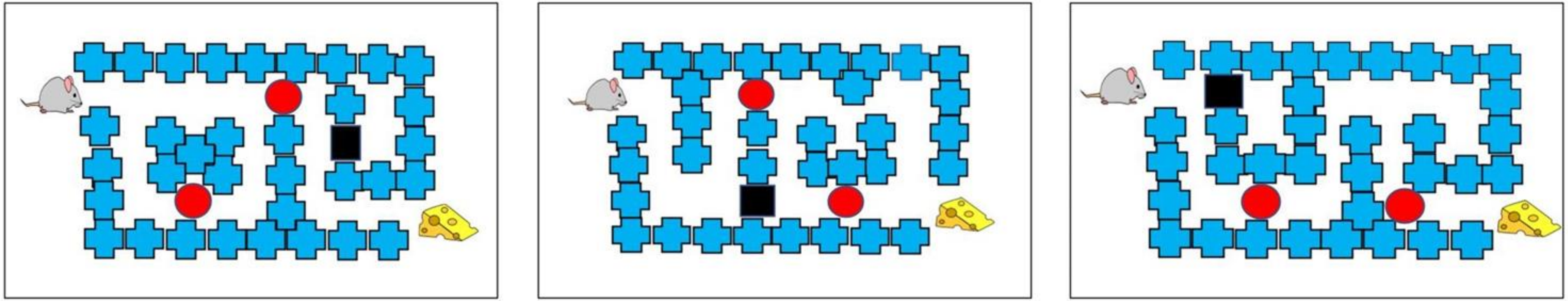

Supplement: Multimedia component 1 [file mmc1.pdf]
