# Supplementary material for: Enhancing computational thinking in early childhood education through ScratchJr integration
Source: Heliyon. 2024 Apr 30;10(10):e30482. doi: 10.1016/j.heliyon.2024.e30482 (PMC11109739; doi:10.1016/j.heliyon.2024.e30482)

Ποιο ΔΕΝ μπορεί να προγραμματιστεί;

Which CANNOT be programmed?

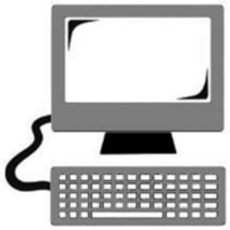

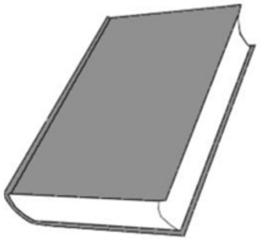

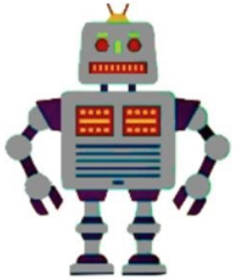

Ποιο λειτουργεί σαν ένας υπολογιστής.

Which works the most like a computer?

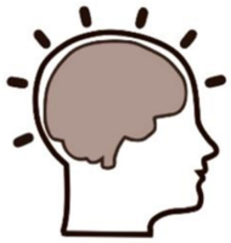

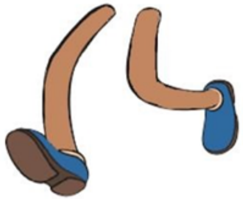

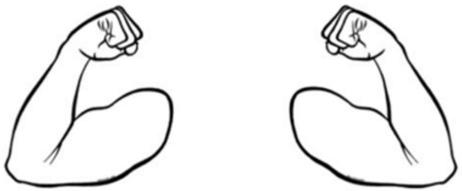

Αυτό το πρόγραμμα ΔΕΝ είναι σωστό. Πώς μπορεί να αλλάξει ώστε η γάτα να τρέξει προς την μπάλα, να την ακουμπήσει και η μπάλα να κυλήσει.

This program is NOT correct. How can it be changed so that the cat runs to the ball, touches it and the ball rolls.

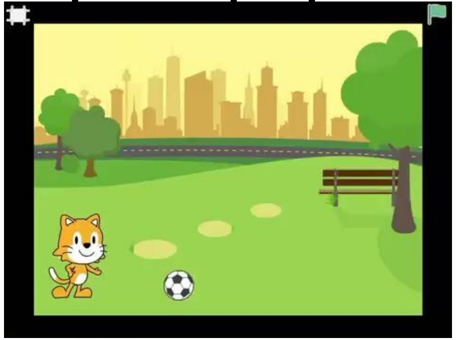

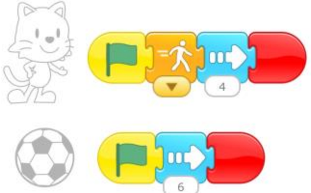

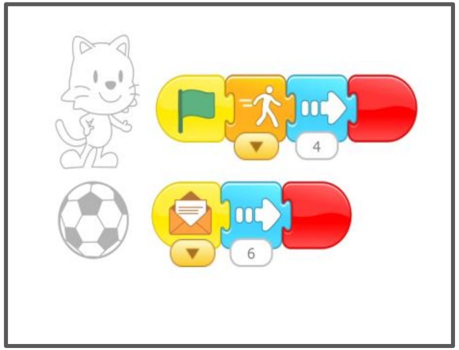

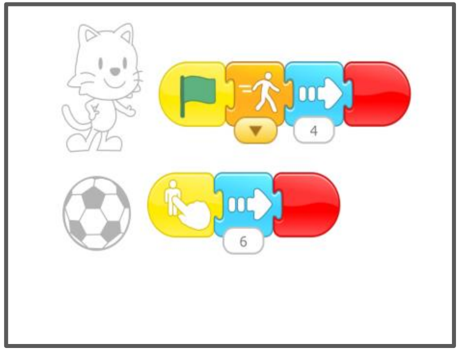

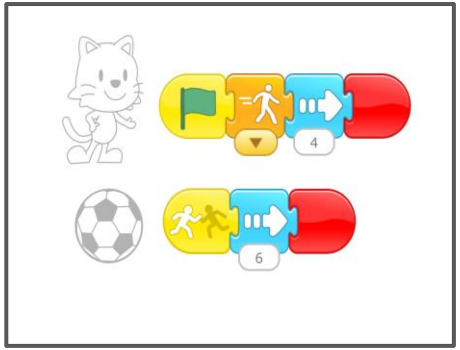

Αυτό το πρόγραμμα δεν είναι σωστό γιατί η γάτα λέει “γεια” 4 φορές. Ποιο πρόγραμμα θα επιλέξω ώστε η γάτα να λέει “γεια” ΜΟΝΟ μία φορά;

This program is not correct because the cat says "hi" 4 times. Which program should I choose so that the cat says "hi" ONLY once?

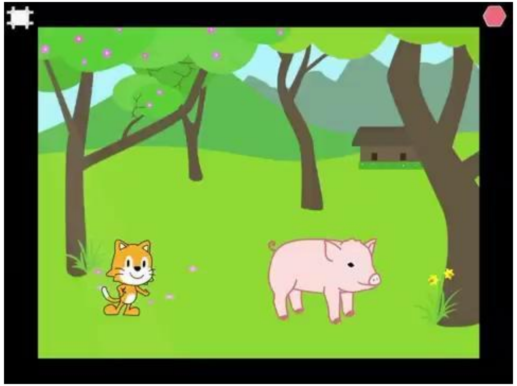

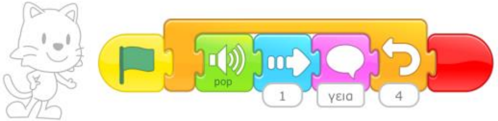

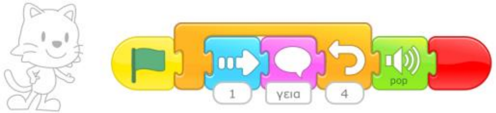

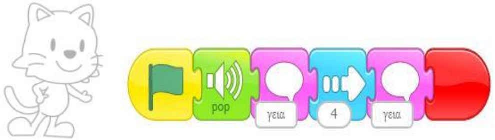

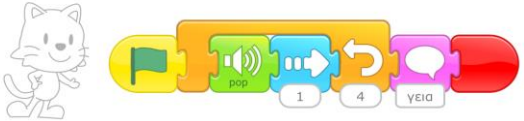

Ποια είναι η σωστή σειρά για να προσθέσεις μία καμηλοπάρδαλη για παρέα στον ελέφαντα.

What is the correct order to add a companion giraffe to the elephant?

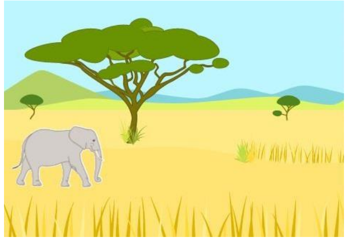

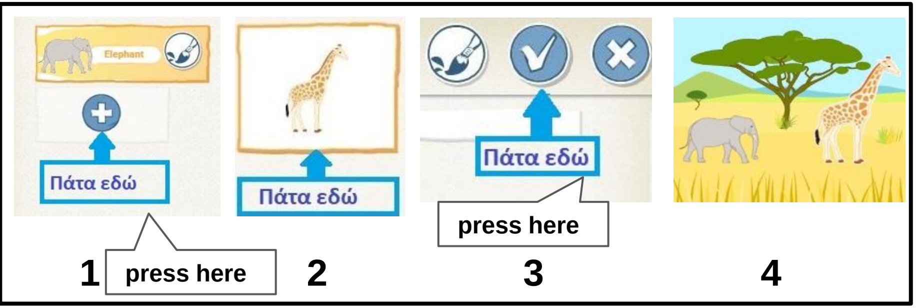

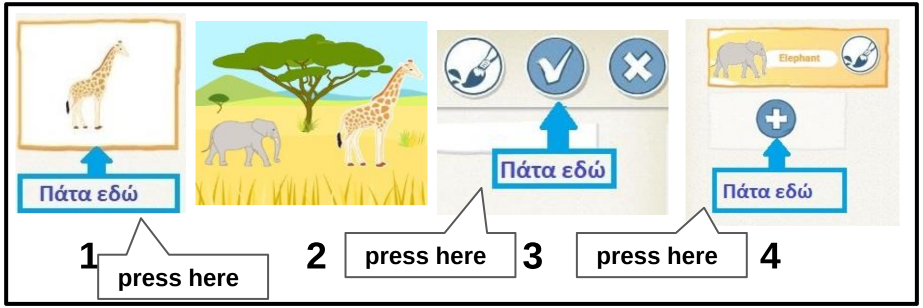

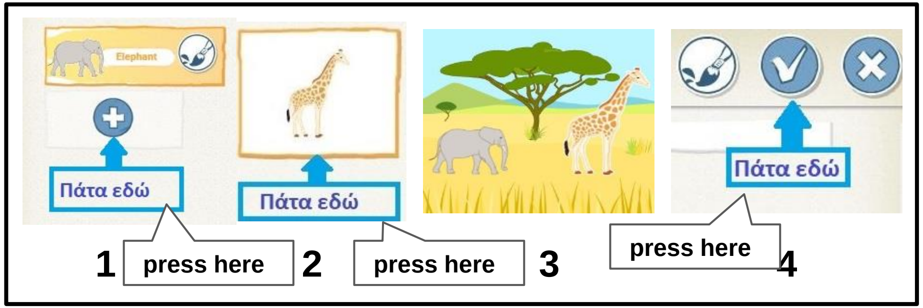

Ποια σχήματα χρειάζεσαι για να φτιάξεις αυτή την πεταλούδα;

What shapes do you need to make this butterfly?

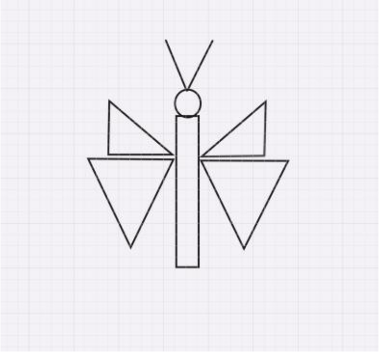

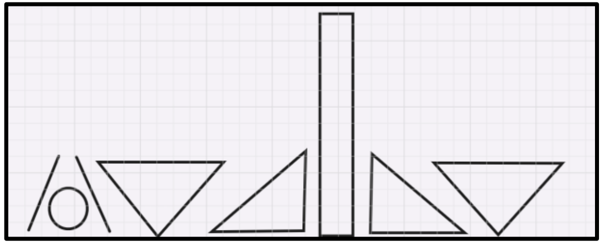

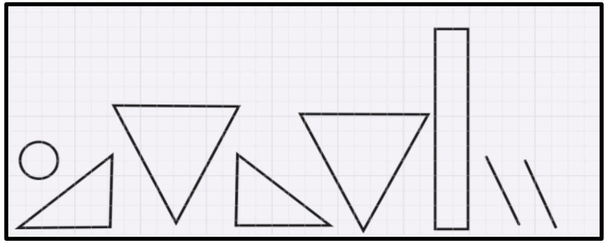

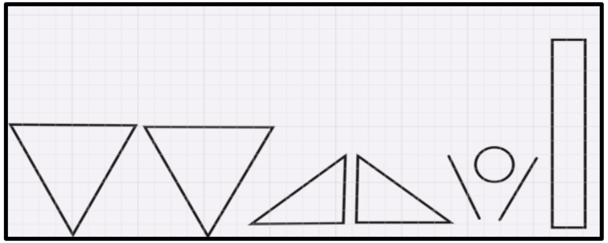

Κύκλωσε τα πλακίδια που θα χρησιμοποιήσεις για να φτιάξεις αυτό το πρόγραμμα.

Circle the blocks you will use to make this program.

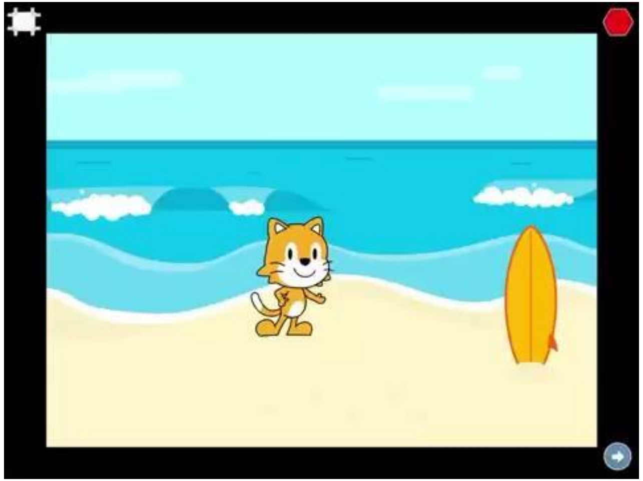

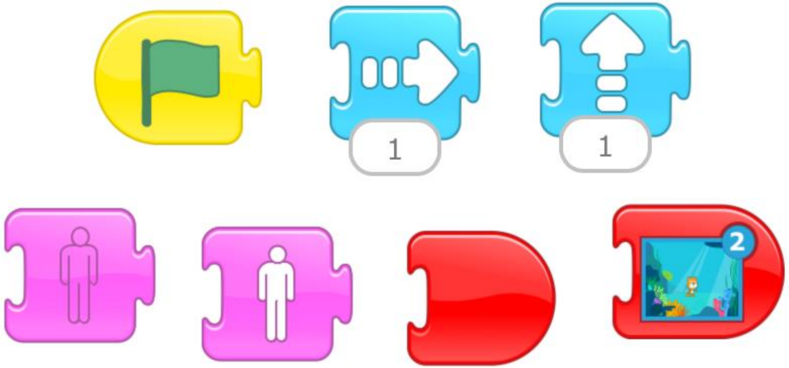

Το γουρούνι πεινάει πολύ. Ποια είναι η πιο γρήγορη διαδρομή για να φάει ένα μήλο.

The pig is very hungry. What is the fastest route to eat an apple?

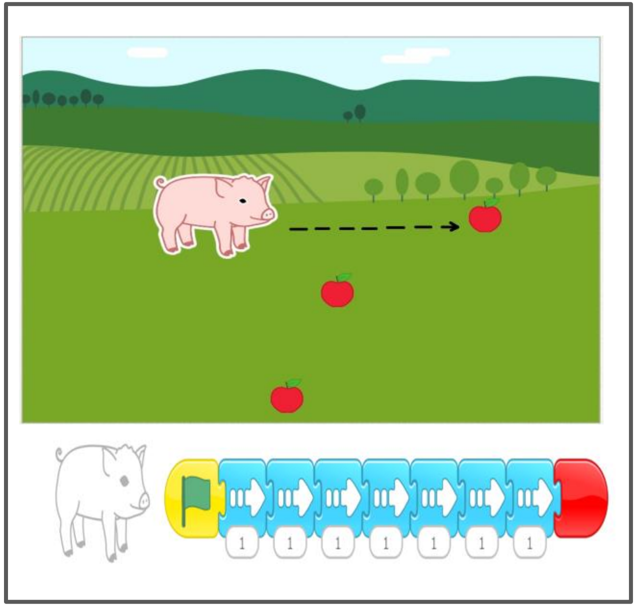

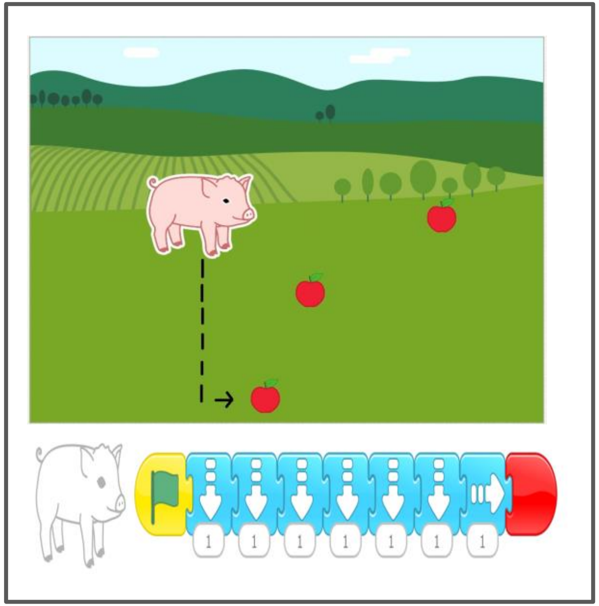

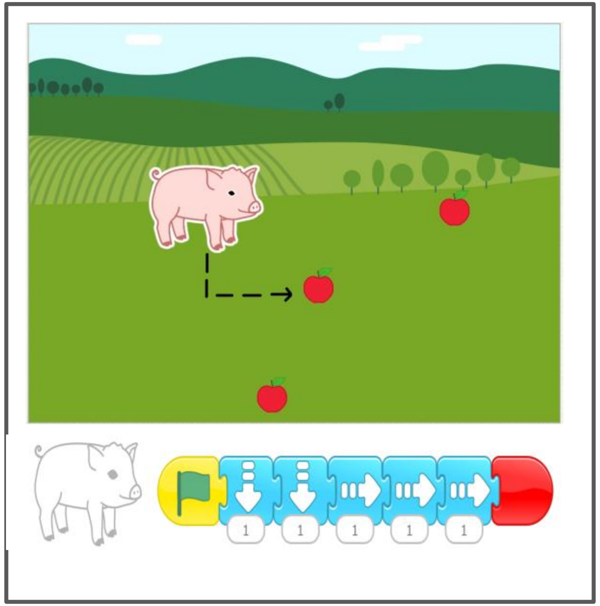

Το γουρούνι πεινάει πολύ. Ποια είναι η πιο γρήγορη διαδρομή για να φάει 2 μήλα.

The pig is very hungry. What is the fastest route to eat 2 apples?

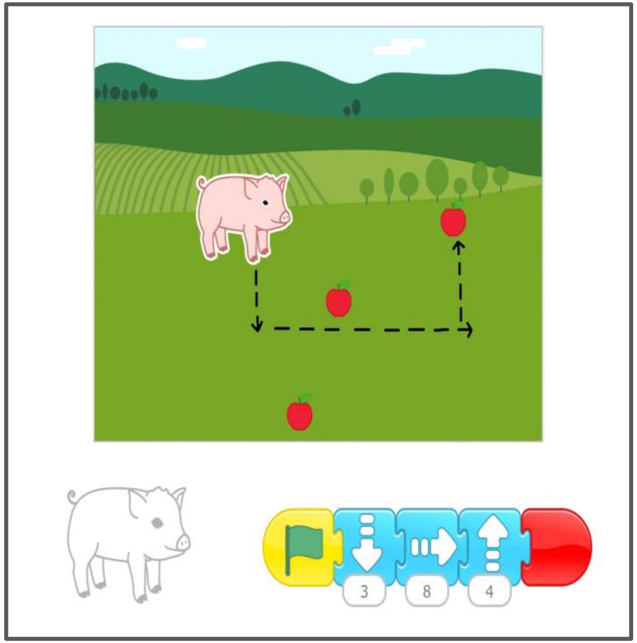

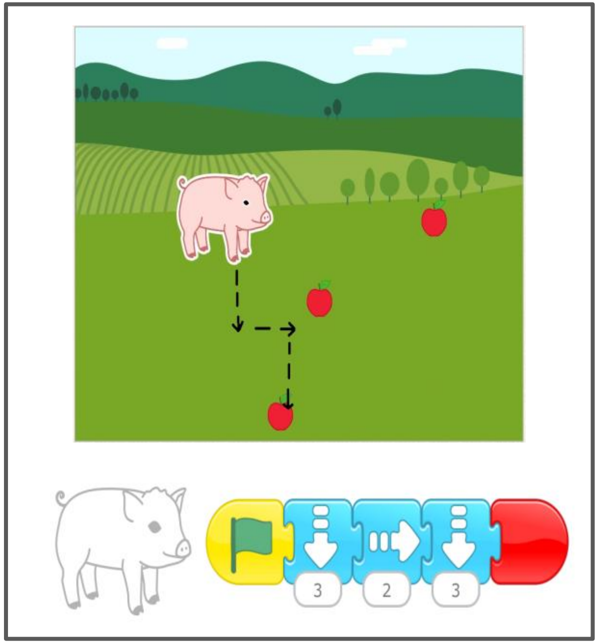

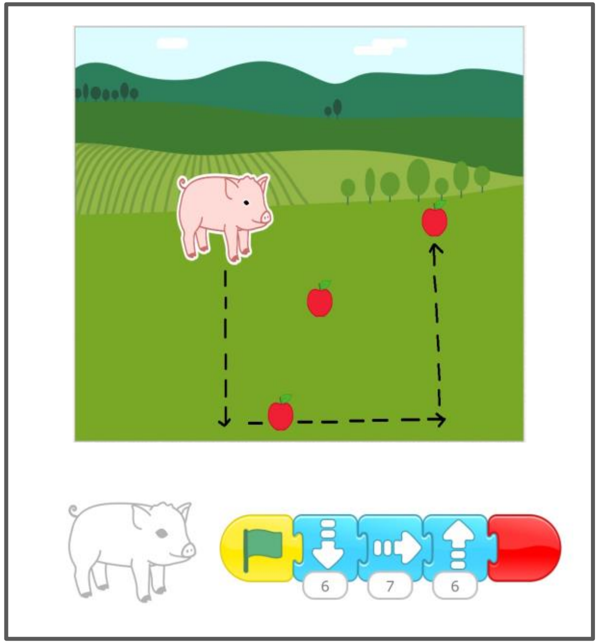

What comes next?

Τι θα ακολουθήσει;

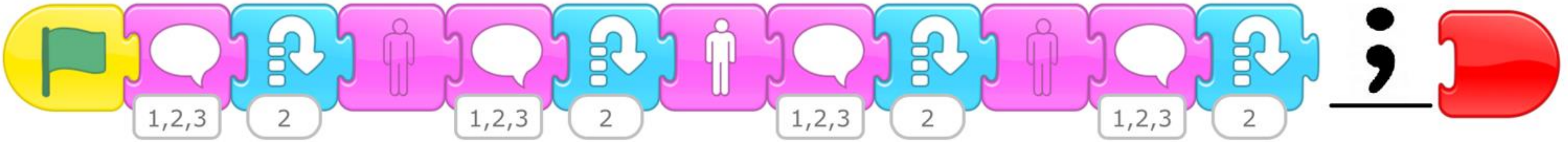

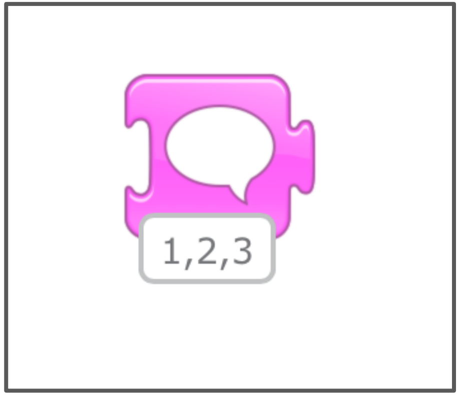

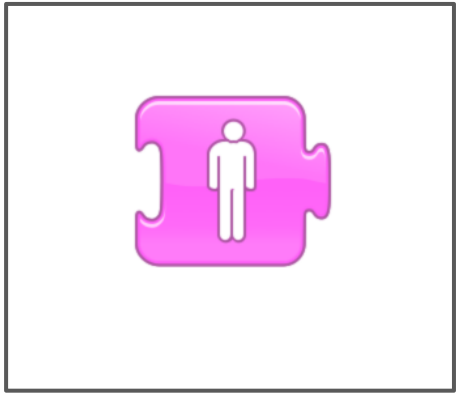

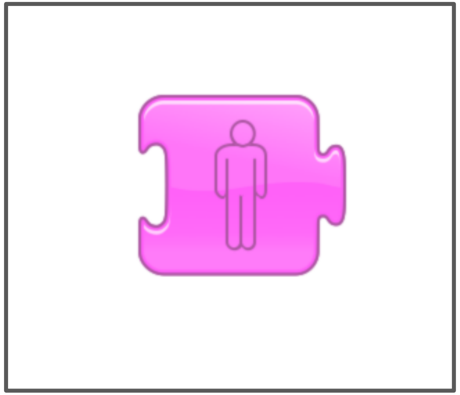

What comes next?

Τι θα ακολουθήσει;

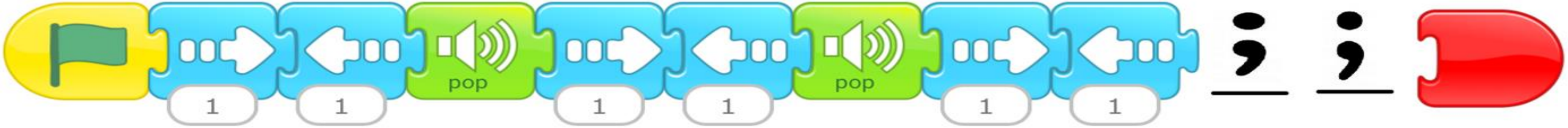

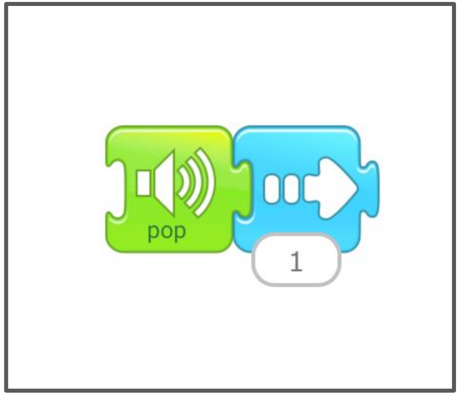

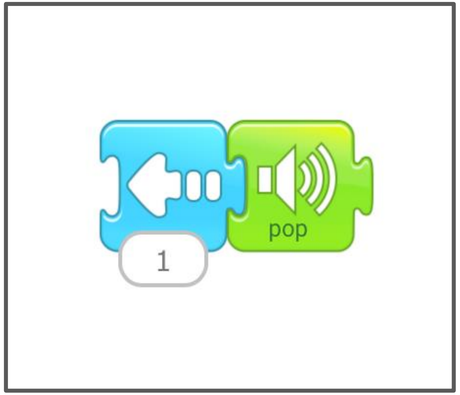

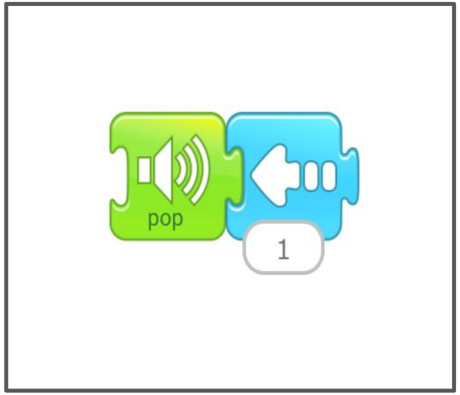

Ο γατούλης ακουμπάει τον σκύλο και ο σκύλος εξαφανίζεται.Ποιο πρόγραμμα θα επιλέξω;

The cat touches the dog and the dog disappears.Which program should I choose?

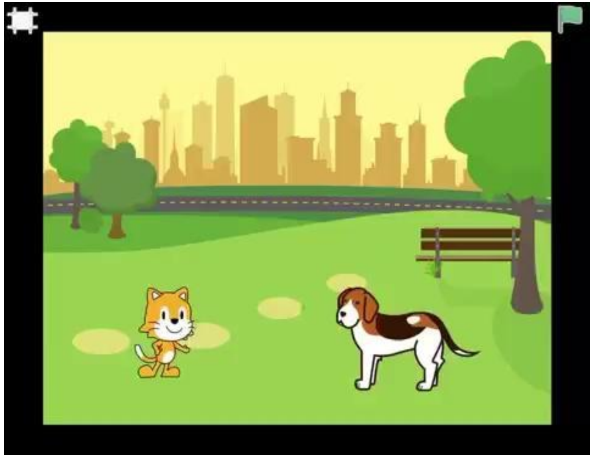

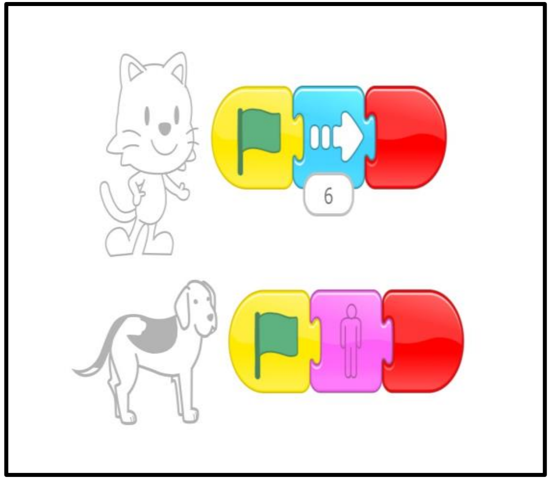

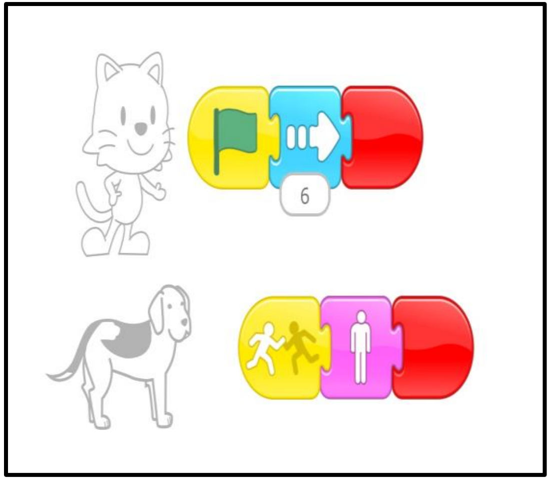

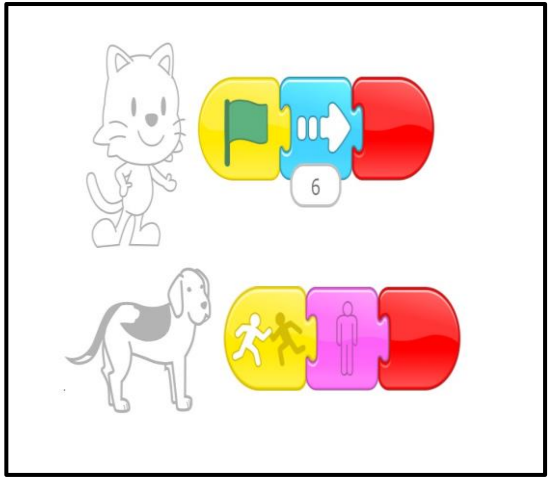

Ποιο πρόγραμμα θα επιλέξω ώστε το άλογο να κερδίσει το γουρούνι;

Which program should I choose so that the horse beats the pig?

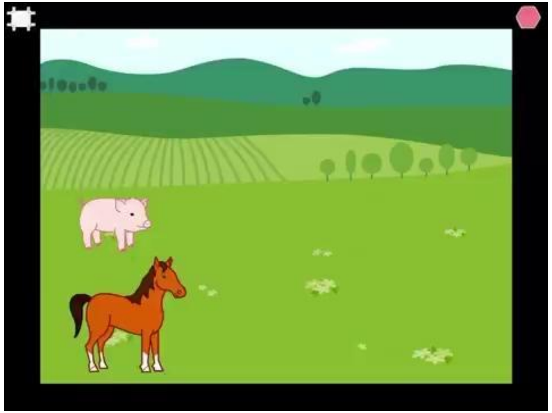

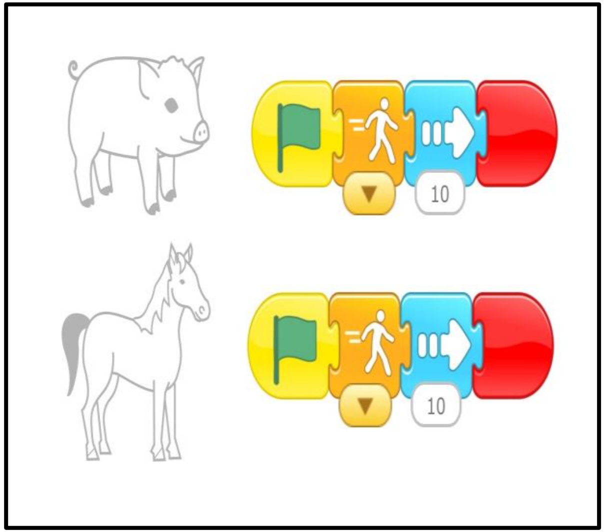

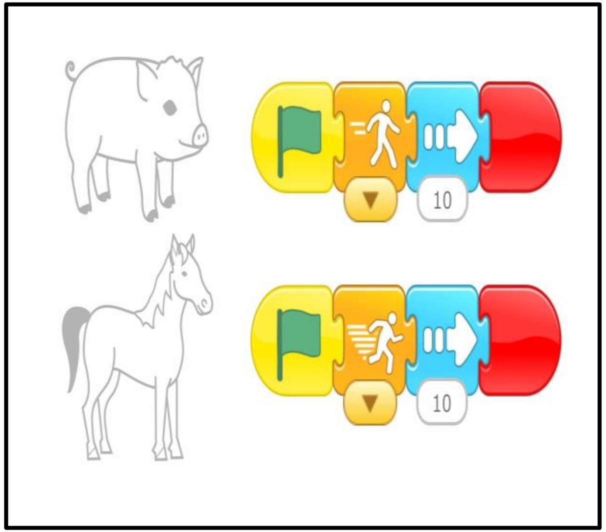

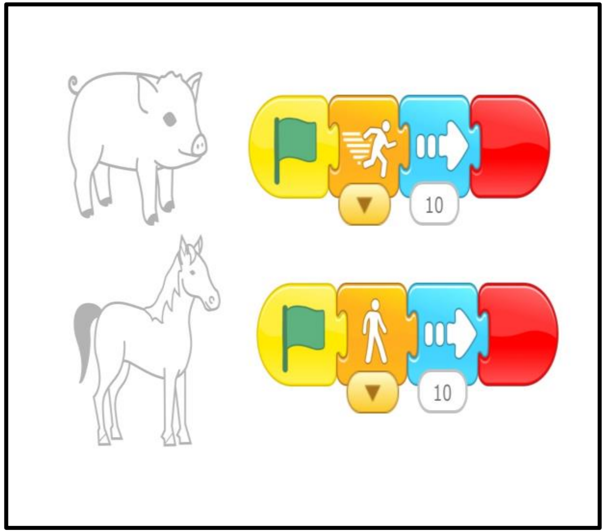

Το ψάρι δεν μπορεί να περάσει τους αστερίες. Ποιο ψάρι θα φτάσει στον φίλο του, το καβούρι;

The fish cannot pass the starfish. Which fish will reach his friend, the crab?

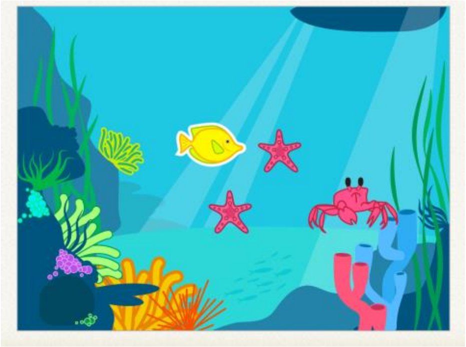

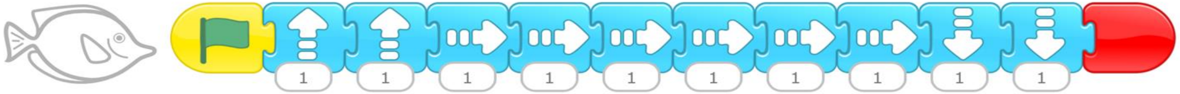

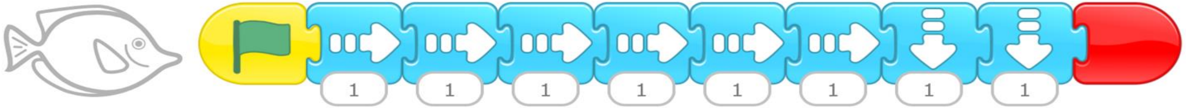

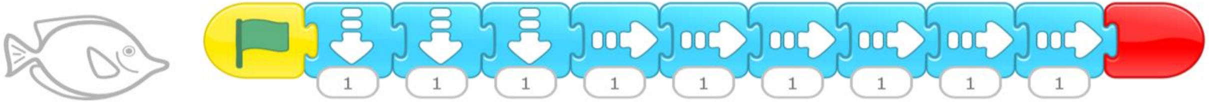

Το κίτρινο ψάρι δεν μπορεί να περάσει τους αστερίες αλλά μπορεί να περάσει από το πράσινο ψάρι. Ποιο ψάρι θα φτάσει στον φίλο του, το καβούρι;

The yellow fish cannot pass the starfish but it can pass the green fish. Which fish will reach his friend, the crab?

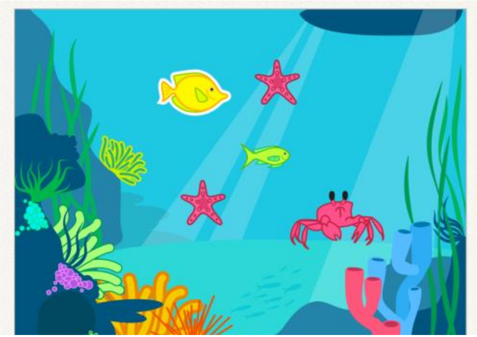

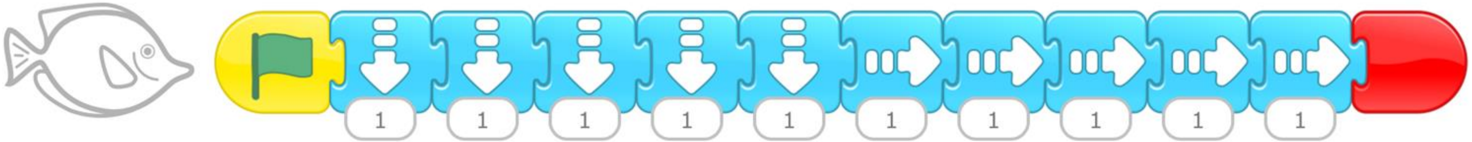

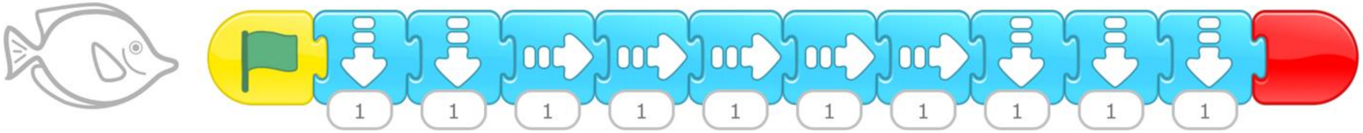

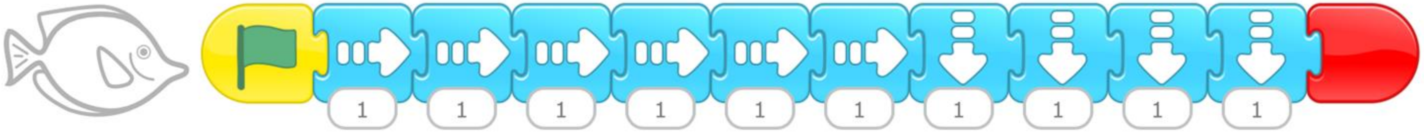

Supplement: Multimedia component 2 [file mmc2.pdf]
